# The role of social determinants of health in the risk and prevention of group A streptococcal infection, acute rheumatic fever and rheumatic heart disease: A systematic review

**DOI:** 10.1371/journal.pntd.0006577

**Published:** 2018-06-13

**Authors:** Pasqualina M. Coffey, Anna P. Ralph, Vicki L. Krause

**Affiliations:** 1 Centre for Disease Control, Department of Health, Darwin, Northern Territory, Australia; 2 Menzies School of Health Research, Darwin, Northern Territory, Australia; 3 Division of Medicine, Royal Darwin Hospital, Darwin, Northern Territory, Australia; QIMR Berghofer Medical Research Institute, AUSTRALIA

## Abstract

**Background:**

Rheumatic heart disease (RHD) poses a major disease burden among disadvantaged populations globally. It results from acute rheumatic fever (ARF), a complication of Group A Streptococcal (GAS) infection. These conditions are acknowledged as diseases of poverty, however the role of specific social and environmental factors in GAS infection and progression to ARF/RHD is not well understood. The aim of this systematic review was to determine the association between social determinants of health and GAS infection, ARF and RHD, and the effect of interventions targeting these.

**Methodology:**

We conducted a systematic literature review using PubMed, the Cochrane Library and Embase. Observational and experimental studies that measured: crowding, dwelling characteristics, education, employment, income, nutrition, or socioeconomic status and the relationship with GAS infection, ARF or RHD were included. Findings for each factor were assessed against the Bradford Hill criteria for evidence of causation. Study quality was assessed using a standardised tool.

**Principle findings:**

1,164 publications were identified. 90 met inclusion criteria, comprising 91 individual studies. 49 (50.5%) were poor quality in relation to the specific study question. The proportion of studies reporting significant associations between socioeconomic determinants and risk of GAS infection was 57.1%, and with ARF/RHD was 50%. Crowding was the most assessed factor (14 studies with GAS infection, 36 studies with ARF/RHD) followed by socioeconomic status (6 and 36 respectively). The majority of studies assessing crowding, dwelling characteristics, education and employment status of parents or cases, and nutrition, reported a positive association with risk of GAS infection, ARF or RHD. Crowding and socioeconomic status satisfactorily met the criteria of a causal association. There was substantial heterogeneity across all key study aspects.

**Conclusion:**

The extensive literature examining the role of social determinants in GAS infection, ARF and RHD risk lacks quality. Most were observational, not interventional. Crowding as a cause of GAS infection and ARF/RHD presents a practical target for prevention actions.

## Author summary

Rates of rheumatic heart disease (RHD) are high in disadvantaged populations globally. It results from acute rheumatic fever (ARF), a complication of Group A Streptococcal (GAS) infection. These are described as diseases of poverty, but exactly what components of poverty promote them has been unclear. The aim of this review was to find what specific social and environmental factors are associated with GAS infection, ARF and RHD, and if actions targeting these can reduce disease rates. We did a search of published literature and found 90 relevant articles. Many supported an association between GAS infection, ARF or RHD and crowding, dwelling characteristics, low education level and employment status, poor nutrition and low social class. There was enough evidence to show that crowding and socioeconomic disadvantage increase the risk of GAS infection and ARF/RHD. However, most studies were of fair to poor quality in their ability to answer the research question, and there was little interventional research. This may relate to challenges inherent in intervening to change social determinants of health, but may also suggest lesser research attention to health issues affecting disadvantaged populations. The association between crowding and disease risk strongly supports initiatives to reduce crowding. This should become a key target for ARF and RHD prevention.

## Introduction

Rheumatic heart disease (RHD) is an important cause of cardiac morbidity and mortality in disadvantaged populations globally [1–4]. It results from acute rheumatic fever (ARF), which itself occurs as an abnormal immunological response to Group A Streptococcal (GAS) infection of the throat (streptococcal pharyngitis) and possibly streptococcal skin infection[5] in susceptible hosts [6]. GAS pharyngitis is spread through direct person-to-person transmission via saliva or nasal secretions [7]. Generally very few people will develop ARF after GAS exposure, but it may be as high as 5 to 6% in certain groups with greater susceptibility and heavy GAS exposure [8]. Recurrences of ARF cause progressive valvular damage, with between 50 and 75% of cases progressing to RHD [8]. RHD is a chronic and debilitating condition characterised by complications such as arrhythmias and heart failure [6]. ARF and RHD most often affect children and young adults [2]. The true burden of the disease is expected to be far higher than the benchmark estimates, but even at conservative calculations, it is equivalent to approximately one quarter of the global DALY burden of cancer [3].

Globally, ARF and RHD are almost exclusively seen in developing nations or among disadvantaged populations within developed nations [2]. Among populations safeguarded by high standards of living, RHD rates are virtually zero. This dramatic contrast highlights the influence of environmental, economic, social and behavioural conditions on risk of GAS infection and progression to ARF and RHD. Despite the role of social determinants of health in disease genesis, key RHD control programs and guidelines[4, 9, 10] do not specifically address primordial prevention. Primordial prevention aspires to establish and maintain conditions to minimize hazards to health. It consists of actions and measures that inhibit the emergence and establishment of environmental, economic, social and behavioural conditions, cultural patterns of living known to increase the risk of disease[11]–that is, strategies that aim to eliminate exposure to risk factors in the first place. Current strategies focus on primary prevention (early detection and treatment of GAS infections), secondary prevention (delivering intramuscular penicillin every four weeks) and tertiary prevention (medical and surgical management of heart failure) [12]. These cornerstones of RHD control are heavily reliant on health care services being available and accessible, a sufficient level of health literacy and appropriate health seeking behaviour among the general population, and ongoing commitment from cases to receive their injections. However, in settings of poverty or marginalisation, these elements cannot be guaranteed. Furthermore, medical treatment and case management can reduce morbidity and mortality, but they will not change the underlying risk to vulnerable populations [13].

Therefore primordial prevention should be part of a comprehensive strategy to eliminate RHD as a public health problem–a global goal [14]. But while ARF and RHD are generally referred to as ‘diseases of poverty’,[1–4] there is uncertainty about what specific aspects of poverty create the conditions that cause RHD. In order to implement evidence-based preventive strategies at the primordial level, more needs to be understood about the relative contribution of the individual components of poverty, such as household crowding, educational attainment, employment, income, nutrition and overall socioeconomic status to RHD risk. Additionally, evidence for any public health interventions targeting these primordial-level factors needs evaluating.

To provide the evidence base for primordial-level preventative interventions to control RHD, we undertook a systematic review with two aims: to determine the association between social determinants of health and GAS infection, ARF and RHD, and to determine the effect on GAS infection, ARF and RHD of interventions targeting these determinants.

## Methods

### Search strategy

We conducted a systematic literature review on the association between socioeconomic and environmental factors and GAS infection, ARF or RHD, and on the impact of interventions targeting these factors.

The search was conducted between August and October 2016 and eligible articles were identified by searching three databases: PubMed, Cochrane Library and Embase. MeSH terms, key words and Emtree terms (Embase thesaurus headings) searches were conducted. See S1 Table for full details of the search strategy.

All titles and available abstracts were screened by one author (PC). Articles were eligible for inclusion in the analysis of interventions if they were in English, reported on an intervention that encompassed: health promotion, education, or behaviour change targeting social determinants of GAS infection (including impetigo), ARF, or RHD; the provision of hygiene hardware, aids or household infrastructure; or household crowding reduction. Interventions could be at an individual, household, health centre, school or community level. Inclusion criteria for observational studies were that they must have reported on at least one socioeconomic or environmental variable and its relationship with GAS infection including impetigo), ARF or RHD incidence or prevalence, measured objectively at an individual, ecologic or population level with a comparison group (either study controls or use of population data e.g. census). Studies were excluded: if they were not in English; assessed pharmacological interventions only; were of very poor quality; or provided only a subjective appraisal of or no description of methods for ascertaining socioeconomic and environmental factors. No restrictions were set with regards to date of publication. Factors specified in the search strategy included: crowding (household or other settings), income, dwelling characteristics, education level, occupation/employment, social class, and nutrition. These were chosen through a scoping scan of selected literature sources informed by the authors’ prior knowledge in this area. There were no limitations on participant age, setting, geographic location or publication date of the studies. References of selected studies were searched for further articles not covered in the primary search strategy.

The primary outcome was reduction in GAS infection, ARF, or RHD from an intervention targeting a socioeconomic or environmental factor. The secondary outcome was evidence of a causal relationship or association of GAS infection, ARF or RHD with specific social determinants of health.

The full text of the articles were reviewed by the same author (PC) and data were extracted to a template that included information on article details (title, year, first author), study type, study methodology, participants and setting, outcomes, and additional notes including salient points from the discussion or authors' conclusions. Study quality and risk of bias pertaining to measurement of determinants and outcomes of interest to this review were assessed for each study using the National Institute of Health Study Quality Assessment Tool,[15] with the appropriate template used based on the study type (S1 Text). Ecologic studies were judged on additional criteria as described in Dufault and Klar (2011) [16]. Study quality as relevant to this review was rated as: very poor (subsequently excluded), poor, fair, fair to good and good.

### Descriptive analysis

Descriptive analysis involved recording the frequency of specific factors measured across the studies and whether a statistically significant association with GAS infection, ARF or RHD was identified, the direction of the association (considered positive if greater degrees of the factor of interest were associated with higher disease rates), mixed, negative, possible or not found. A p value of <0.05 was used to define a statistically significant association. ‘Possible’ designated a reported positive association where a test of significance was not provided, nor means to calculate one. ‘Mixed’ designated a combination of positive, negative or absence of association for different measures of the same variable. Results for GAS infection were presented separately and ARF/RHD to reflect the different epidemiology and natural history of the conditions.

The findings were synthesised in the context of study quality and strengths and limitations of findings. Systematic reviews (n = 4) were reviewed for quality and findings separately.

The evidence of the link between each of the main factors explored and GAS infection, ARF and RHD was assessed against the Bradford Hill criteria, a set of nine guiding principles for interpreting causal relationships between environmental influences and disease. The criteria are: strength of association, consistency, temporality, biological gradient, plausibility, coherence, experiment, specificity and analogy [17].

### Statistical analysis

The studies were too heterogeneous in exposure and outcome measures for meta-analysis.

In some instances where statistical testing was not performed in the original study, but enough data were presented to allow analysis to be undertaken (i.e. a 2 X 2 table with denominators), simple tests of significance (chi-squared, odds ratio (OR) with 95% confidence intervals, or relative risk (RR)) were calculated using the Tables for Epidemiologists function in Stata 13.1 (Statacorp, Texas 2013). These additional analyses are indicated in S2–S8 Tables.

Statistical analysis was conducted to check for correlation of study type, quality of study (fair or greater), outcome of interest (GAS infection, ARF or RHD) and positive outcomes using chi-squared and Fisher’s exact tests of significance. Correlation between year of publication and study quality was analysed using binomial variables of published in previous 20 years (>1996) and study quality equal to or greater than fair. Analysis was carried out using Stata 13.1 (Statacorp, Texas 2013).

This review adheres to the Preferred Reporting Items for Systematic Reviews and Meta-Analyses (PRISMA) checklist (S1 Checklist) [18].

## Results

### Overview

The search strategy identified 1,164 articles in PubMed, 273 articles in Embase and 84 articles in the Cochrane Library (S1 Fig). An additional 21 study articles were recovered from manual reference searches of the selected articles. Of these 114 were selected on the basis of title and abstract. The full text was unavailable for eight articles. The full texts of the remaining articles were reviewed and of these one article was excluded due to being very poor quality as were two studies from an article [19] of three studies were excluded. Hence 90 articles met the inclusion criteria comprising 91 individual studies included in the general analysis, as one article contained two separate studies (Table 1) [20].

**Table 1:** Summary of included studies with outcome of GAS infection, including findings, study type and quality assessment.

	Crowding	Dwelling characteristics and facilities	Education	Employment	Income	Nutrition	Socioeconomic status
**Number of studies, n**	14	7	1	0	3	0	6
**Association with GAS infection, n (%)**							
**Positive**	6 (42.9)	4 (57.1)	0 (0.0)	NA	1 (33.3)	NA	3 (50.0)
**Mixed**	2 (14.3)	0 (0.0)	0 (0.0)		1 (33.3)		0 (0.0)
**Possible**	1 (7.1)	0 (0.0)	0 (0.0)		1 (33.3)		0 (0.0)
**No association**	5 (35.7)	3 (42.9)	1 (14.3)		0 (0.0)		3 (50.0)
**Study type, n (%)**							
**Case control**	1 (7.1)	0 (0.0)	0 (0.0)	NA	0 (0.0)	NA	0 (0.0)
**Case series**	1 (7.1)	0 (0.0)	0 (0.0)		0 (0.0)		0 (0.0)
**Cohort**	6 (42.9)	2 (28.6)	0 (0.0)		1 (33.3)		2 (33.3)
**Control trial**	1 (7.1)	0 (0.0)	0 (0.0)		0 (0.0)		0 (0.0)
**Cross section**	4 (28.6)	2 (28.6)	1 (14.3)		2 (66.7)		2 (33.3)
**Ecologic**	1 (7.1)	0 (0.0)	0 (0.0)		0 (0.0)		2 (33.3)
**Randomised control trial**	0 (0.0)	3 (42.9)	0 (0.0)		0 (0.0)		0 (0.0)
**Quality assessment, n (%)**							
**Good**	1 (7.1)	1 (14.3)	0 (0.0)	NA	0 (0.0)	NA	1 (16.7)
**Fair to good**	0 (0.0)	0 (0.0)	0 (0.0)		0 (0.0)		1 (16.7)
**Fair**	2 (14.3)	1 (14.3)	0 (0.0)		0 (0.0)		1 (16.7)
**Poor to fair**	3 (21.4)	2 (28.6)	0 (0.0)		1 (33.3)		2 (33.3)
**Poor**	8 (57.1)	3 (42.9)	1 (14.3)		2 (66.7)		3 (50.0)

Several articles were based on the same study population. Two articles by McDonald et al.(2006) [21] (2008) [22] shared the same participants and a third, a subset of these [5]. The articles authored by Adanja et al.(1988) [23] (1991) [24] and Vlajinac et al. (1989) [25] (1991) [26] utilised the same participants, as did two studies by Zaman(1998a) [27] (1998b) [28]. Four systematic reviews were analysed separately [2, 29–31].

33 studies reported ARF as the outcome, 27 reported RHD, 10 reported both, and 21 reported GAS infection. There were 16 case control studies, [23–28, 32–41] nine case series, [42–50] 12 cohort studies, [5, 21, 51–61] one control trial, [62] 28 cross section studies, [19, 63–89] 22 ecologic studies, [20, 90–105] and three randomised controlled trials (RCT) [106–108]. Studies were conducted in 27 countries with two studies including data from multiple countries.

The quality of the studies in design or ability to answer the research question was rated as poor in 45 (50.5%). Only three studies graded as being of good quality. Studies that were case series were most likely to be of poor quality. Two of the three RCTs, seven of 16 (43.8%) case control, and five of 12 (41.7%) cohort studies were of at least fair quality. Studies published after 1996 (25 of 37) were more likely to be of fair or better quality than those published earlier (6 of 54) (OR 16.7, 95% CI 5.59 to 49.71). Study quality was not associated with outcomes.

12 studies (57.1%) reported a positive relationship between at least one socioeconomic factor and risk of GAS infection, and 35 studies (50.0%) reported a positive relationship between a socioeconomic factor and risk of either ARF or RHD.

The likelihood of positive findings was not associated with publication after 1996 or study quality. 100% of the case control studies reported at least one positive association of a socioeconomic factor with disease rates compared to 64.9% of other studies (Fisher’s exact p = 0.002).

### Crowding

50 studies assessed the association between crowding and GAS infection (14 studies), ARF (16 studies), RHD (15 studies) or a combination (5 studies) (Tables 1 and 2; S2 Table). The most common measures of crowding were: persons per household, room, bedroom or bed; number of children or siblings; dwelling space; and sleeping space per person. 12 of 21(57.1%) studies noted a positive association between crowding and the different outcomes of GAS infection, 9 of 16 (56.3%) with ARF, and9 of 15 with RHD (60.0%) and combinations thereof (60.0%), though only 14 found consistent associations across all measures of crowding (42.9% among studies of GAS infection and 30.6% among ARF/RHD). A further 10 studies reported a possible association between crowding and GAS infection, ARF or RHD risk but did not provide a test of significance nor means to calculate one.

**Table 2:** Summary of included studies with outcome of ARF and/or RHD, including findings, study type and quality assessment.

	Crowding	Dwelling characteristics and facilities	Education	Employment	Income	Nutrition	Socioeconomic status
**Number of studies, n**	36	19	16	15	18	15	36
**Association with ARF/RHD, n (%)**							
**Positive**	11 (30.6)	6 (31.6)	4 (21.1)	5 (33.3)	6 (33.3)	4 (26.7)	16 (44.4)
**Mixed**	10 (27.8)	6 (31.6)	6 (31.6)	3 (20.0)	1 (5.6)	8 (53.3)	2 (5.6)
**Possible**	9 (25.0)	1 (5.3)	0 (0.0)	2 (13.3)	2 (11.1)	1 (6.7)	10 (27.8)
**No association**	6 (16.7)	6 (31.6)	6 (31.6)	5 (33.3)	9 (50.0)	2 (13.3)	8 (22.2)
**Study type, n (%)**							
**Case control**	12 (33.3)	10 (52.6)	9 (47.4)	7 (46.7)	9 (50.0)	7 (46.7)	4 (11.1)
**Case series**	3 (8.3)	1 (5.3)	1 (5.3)	0 (0.0)	2 (11.1)	1 (6.7)	4 (11.1)
**Cohort**	3 (8.3)	2 (10.5)	1 (5.3)	3 (20.0)	1 (5.6)	1 (6.7)	1 (2.8)
**Control trial**	0 (0.0)	0 (0.0)	0 (0.0)	0 (0.0)	0 (0.0)	0 (0.0)	0 (0.0)
**Cross section**	11 (30.6)	5 (26.3)	2 (10.5)	3 (20.0)	2 (11.1)	5 (33.3)	14 (38.9)
**Ecologic**	7 (19.4)	1 (5.3)	3 (15.8)	2 (13.3)	4 (22.2)	1 (6.7)	13 (36.1)
**Randomised control trial**	0 (0.0)	0 (0.0)	0 (0.0)	0 (0.0)	0 (0.0)	0 (0.0)	0 (0.0)
**Quality assessment, n (%)**							
**Good**	1 (2.8)	0 (0.0)	0 (0.0)	0 (0.0)	1 (5.6)	0 (0.0)	1 (2.8)
**Fair to good**	4 (11.1)	4 (21.1)	3 (15.8)	1 (6.7)	3 (16.7)	3 (20.0)	1 (2.8)
**Fair**	10 (27.8)	3 (15.8)	5 (26.3)	6 (40.0)	3 (16.7)	5 (33.3)	11 (30.6)
**Poor to fair**	6 (16.7)	3 (15.8)	3 (15.8)	3 (20.0)	2 (11.1)	2 (13.3)	4 (11.1)
**Poor**	15 (41.7)	9 (47.4)	5 (26.3)	5 (33.3)	9 (50.0)	5 (33.3)	19 (52.8)

^1^Note studies may appear more than once if they assessed more than one factor e.g. crowding and education.

Of the 11 studies that assessed crowding after adjusting for one or more independent variables such as household income, seven noted a residual association between crowding and the outcome of interest. Two case control and three ecologic studies demonstrated a continuous effect gradient between crowding and risk of GAS infection, [38] ARF, [41, 90, 109] and RHD [96].

The remaining 11 (22%) studies that examined an association between crowding and disease rates did not demonstrate any association between crowding and disease risk.

### Dwelling characteristics and facilities

Dwelling characteristics and facilities were assessed across a variety of measures such as general housing condition, construction type, specific characteristics (e.g. dampness, ventilation), or facilities (e.g. water, electricity, toilets) in relationship to GAS infection in seven studies and ARF/RHD in 19 (Tables 1 & 2; S3 Table). Four of seven studies reported positive or mixed associations between dwelling characteristics or facilities with increased GAS infection rates, eight of nine with ARF, two of eight with RHD, and both of the studies that assessed ARF and RHD together. General poor condition or standard of housing was associated with increased risk of ARF or RHD in five of nine studies and type of housing construction or material was associated with GAS infection, ARF or RHD risk in six of nine. Home dampness was associated with ARF in five of seven studies (three of these analysed the same population). There were no clear trends among other specific housing characteristics and facilities (e.g. electricity, kitchen facilities, light, potable water, sewerage, and ventilation) and GAS infection or ARF/RHD.

Three diverse RCTs assessed interventions aimed to induce or reduce acquisition of GAS or its sequelae through common household fomites [106, 108] or hygiene practices [107]. Of these only Luby et al’s (2005) RCT demonstrated an effect on GAS related disease (impetigo). Over 4,600 children were randomised to receive hand washing promotion and antibacterial soap, hand washing promotion and plain soap, or no intervention. The mean impetigo incidence was 36% and 24% lower among the antibacterial and plain soap groups compared to the control group respectively.

### Education

The education level of mothers, fathers and cases themselves were explored across 17 studies, with 16 studies reporting ARF/RHD as the outcome (Tables 1 & 2; S4 Table). Low maternal education or literacy was positively associated with ARF incidence in two of three studies, though these two studies shared the same population [23, 25]. Two studies [26, 46] reported results of multivariable analyses and found that maternal education remained associated with ARF after adjusting for other variables. Low maternal literacy was associated with both ARF and RHD in another study [35] and RHD prevalence in one [56] of two studies, [33] but this relationship did not hold in multivariate analysis. There were no clear trends for education levels of fathers, parents combined, or of cases with any of the outcomes.

### Employment

The relationship between employment and ARF/RHD was explored in 15 studies. No studies examined relationship between employment and risk of GAS infection (Tables 1 & 2; S5 Table). Maternal employment was considered a marker of social or economic *disadvantage* rather than advantage in four studies. In these studies, maternal employment was positively association with ARF in two of two studies, [23, 41] and with both ARF and RHD in another study, [35] but was not associated with RHD risk in Mirabel et al’s study (2015) [56]. In contrast, a Fijian based case control study measured maternal employment as a marker of socioeconomic *advantage* and found maternal unemployment was associated with RHD prevalence [33]. There were no associations in any of the five studies that assessed paternal employment [33, 35, 41, 54, 65]. Employment status (unemployment/low class occupation) of the case was found to be associated with RHD in three [40, 61, 76] of four studies [72].

Findings of the three studies that reported GAS infection risk and low income were diverse (Table 1; S6 Table). For ARF/RHD, six of 18 studies reported a positive relationship with low income, while nine demonstrated no association (Table 2). In three studies that undertook multivariate analyses, a positive association was retained in one, [90] but lost in two [36, 40].

The method of assessing income (strata, a binomial measure or comparison of mean income) did not affect the likelihood of reporting an association.

### Nutrition

15 studies (covering 12 separate study populations) assessed the relationship between nutrition and ARF or RHD using either dietary intake or anthropometric measures (Tables 1 & 2; S8 Table). Four (26.7%) reported only significant associations between nutritional impairment and ARF or RHD, though tended to only report one simple measure (e.g. low weight or BMI) [26, 74]. A further eight (53.3%) studies demonstrated mixed results.

### Socioeconomic status

The relative social position of cases compared to non-cases or to the general population was based on geographical, economic, occupational and social factors, or was based on the ownership of specific assets. Socioeconomic status (SES) or social class was assessed in six studies reporting GAS infection as an outcome, and 36 studies reporting ARF/RHD, with three (50%) and 16 (44.4%) respectively showing a definite association (Tables 1 & 2; S8 Table). A further 10 studies showed a possible association between lower social class with ARF/RHD (i.e. studies that did not provide a statistical test of the apparent relationship between ARF and RHD); nearly all had ecologic designs. 10 studies were able to demonstrate a clear gradient of social class and GAS infection,[20] ARF[87, 90] or RHD[63, 67, 71, 77, 98, 110] risk, with several others suggestive of a gradient.

### Systematic reviews

Four systematic reviews of risk factors for GAS infection, ARF or RHD were identified [2, 29–31]. Only one used a systematic search method for social and environmental risk factors for GAS infection, ARF or RHD [29] and none identified the number of studies found for inclusion in the present review. All four reviews explored SES or poverty in some way. In addition, Kerdemelidis et al (2010) [29] and Steer et al (2002) [30] reviewed crowding, nutrition, and housing factors. The quality of the four studies ranged from poor to fair according to our criteria for measurement of determinants and outcomes of interest to this review, and no review produced firm conclusions.

### Factors assessed against the Bradford Hill Criteria

Each socioeconomic or environmental factor was assessed against the Bradford Hill criteria to establish whether a causal relationship with RHD and its antecedents was supported by the evidence contained in this review (Table 3). Crowding provided a sufficient weight of evidence across the criteria to support a causal relationship, as did socioeconomic status to a lesser extent.

**Table 3:** Bradford Hill criteria for evidence of causation as applied to the relationship of GAS infection, ARF and RHD with social determinant factors.

Criteria	Crowding	Dwelling characteristic and facilities	Education	Employment	Income	Nutrition	Socioeconomic status
Strength	Between 1.7 and 2.8 fold risk across various measures of crowding	Between 2.3 and 3.5 fold risk with dampness; 1.8 to 3.6 fold risk with poor construction/material type. Small number of studies reporting strength for other measures	Between 1.7 and 3.9 higher odds of disease with low maternal literacy	Small number of studies reporting strength with wide distribution	Small number of studies reporting strength	Between 1.4 and 2.7 fold risk for underweight. Small number of studies reporting strength for other measures	Range between 1.5 and 5 fold risk among lowest SES compared to middle and high SES groups
Consistency	29/50 (58%) reported positive associations across many study types	16/26 (61.5%) reported positive associations	No	No	No	No	Yes: when clear measures of SES assessed
Temporality	Yes: factor likely to be longstanding	Yes: factor likely to be longstanding	Yes: education of mother or father precedes life of child	Yes: for employment of parents, not necessarily for case	Yes: factor likely to be longstanding	Unknown: Chronic RHD could contribute to poor growth in children.	Yes: factor likely to be longstanding
Biological gradient	Yes: 5 studies	No	No	No	Yes: 1 study	No	Yes: 10 studies
Plausibility	Yes: GAS spread via close contact	No plausible mechanisms discussed for dwelling characteristics. Use of soap is an effective means for removing microorganisms from hands and body.	Yes: maternal literacy impacts on hygiene practices, care-seeking and treatment behaviours	Indirectly: unemployment/lower class employment relates to income, access to health care etc.	Yes: income impacts on access to health care services and material conditions	Yes: nutritional deficiencies are associated with poor immune response to infection	Indirectly: social class relates to income, access to healthcare and living conditions.
Coherence	Yes	Insufficient evidence	Yes	Insufficient evidence	Yes	Yes	Yes
Experiment	No	Mixed results-soap reduced impetigo; fomite intervention did not change GAS acquisition. Nil for dwelling characteristics	No	No	No	Yes-very poor quality	No
Specificity	No	No	No	No	No	Dietary supplementation with eggs was reported to be associated with lower ARF case numbers	No
Analogy	Meningococcal disease [111–113] and respiratory syncytial virus [114–116]	Housing improvements improve respiratory health[117]	Increased maternal literacy associated with decreased child diarrhoeal disease, fever and respiratory infection [118]	Higher adult mortality rate among lower employment grades [119]	Adult mortality rate and income [120]	Childhood malnutrition has strong relation with risk of death from diarrhoeal and acute respiratory infections [121]	Higher odds of neglected tropical diseases among socioeconomically disadvantaged groups[122]

ARF: Acute rheumatic fever GAS: Group A Streptococcus SES: Socioeconomic status

The strengths of the observed relationships of crowding and outcomes were modest; odds ratio or relative risk calculations for binary outcomes (i.e. crowded vs. not crowded whether at a bed, bedroom or housing level) produced around a twofold likelihood or risk of GAS infection, ARF and RHD. In Jaine et al’s ecologic study of ARF cases across New Zealand, after adjusting for average income and number of children aged 5–14 years, the authors noted that a 1% increase in the proportion of households defined as being crowded conferred a 6.5% increase in the expected ARF count at a census area unit level [90].

Regarding consistency, 29 of the 50 studies that assessed crowding reported statistical evidence of an association with GAS infection, ARF or RHD risk. Further studies demonstrated an apparent relationship but did not conduct any significance testing.

The necessary criteria of temporality between cause and effect was met.

While an individual may experience housing instability or change, the characteristics relevant to crowding, e.g. amount of bedrooms a family can afford, is not likely to change greatly during an individual’s childhood [23, 98]. Therefore a point in time capture of data (as in a cross-sectional study) would be likely to represent the living conditions that a case was subject to prior to the development of the condition. As previously described 5 studies demonstrated a gradient in the relationship between crowding and GAS infection, ARF or RHD [38, 41, 90, 96, 109].

The biological mechanism by which crowding exerts its effect on ARF and RHD is via its relationship to GAS acquisition. Crowding fosters intimate contact and consequent GAS transmission directly by human-to-human contact and via droplet spread [123]. Higher GAS infection rates increase the chance that from any one event ARF sequelae will develop. The role of household crowding as the chief driver of GAS transmission was demonstrated in Levine et al’s large cohort study (1966) [38]. Here, GAS infections were not randomly distributed throughout the cohort but clustered within discrete family units as evidenced by high serological concordance between positive family members. The cause and effect interpretation of crowding and GAS acquisition coheres with our knowledge of the natural history and biology of these conditions.

There were no experimental studies assessing crowding and GAS infection, ARF or RHD risk. The closest such evidence comes from findings of an US Air Base Streptococcal Laboratory [50]. This group reported higher acquisition rates of GAS in new army recruits relating to the vicinity of their bed to a known GAS carrier and the amount of carriers within each barracks. In this study, rates of GAS acquisition in new (unexposed) recruits was documented in relation to their distance from the untreated colonised index case, showing a gradient effect; closer proximity was associated with higher risk, giving the study an almost quasi-experimental design. However, this study failed to test whether an intervention against crowding (i.e. actively moving beds further away) was an effective means of decreasing GAS transmission.

Crowding does not exhibit specificity for GAS infection and its sequelae. It is an established health risk for transmissible diseases, especially of those with epidemic potential where outbreaks are more frequent and more severe when the population density is high [124]. An analogy to the droplet transmission of GAS and its predilection among children are offered by the firm observations of the link between crowding and meningococcal infection[111–113] and respiratory syncytial virus [114, 115, 125].

Socioeconomic status also compared favourably against the Bradford Hill criteria, however the causal relationship is exerted through the influence of intermediary factors (e.g. crowding, income, education), which individually do not carry the same weight of evidence. A gradient of higher disease risk with lower socioeconomic status was demonstrated across ten studies.

## Discussion

This systematic review identified 91 studies spanning 80 years that have assessed the relationship of social and environmental factors of crowding, dwelling characteristics and facilities, education, employment, income, nutrition and socioeconomic status with GAS infection, ARF, and RHD. Nearly all studies were observational rather than intervention studies. Crowding was the most frequently assessed factor followed by socioeconomic status. The majority of studies that assessed a measure of crowding and risk of GAS infection, ARF, or RHD reported a positive association with crowding; as did those examining dwelling characteristics, education levels and employment status of parents or cases, and nutrition. However, there was considerable heterogeneity in measures used, study settings and outcome ascertainment.

We noted a lack of well-designed research and interventions aimed at unravelling poverty as a mechanism for ARF and RHD, which is at odds with the widespread acceptance of ARF and RHD as diseases of poverty [2]. This paradox is exemplified in the hopeful remarks of Perry et al (1937)—the earliest study included in this review- that the noted association between crowding and ARF be ‘the starting-point and not the end of research…and that [further] research…may be fruitful in elucidating the aetiology of rheumatic heart disease and in discovering means for its prevention’; [96] yet 80 years later there remains a paucity of evidence of preventative actions at the primordial level.

This issue is well described among the neglected tropical diseases, [13, 122, 126, 127] a diverse group of communicable diseases that cause significant burden of suffering and economic impacts among poor and marginalised populations living in tropical and subtropical regions [128]. Poverty creates the milieu for these diseases to flourish; the low resource settings then exacerbate difficulties inherent in conducting high quality observational or interventional research. Finally, the neglect of the social, economic, political and physical contexts in which affected populations live, leaves the root causes unchanged [13, 122].

Given the study designs and limited quality of papers, we used the Bradford Hill criteria, a set of guiding principles for interpreting links between environmental influences and disease, as a pragmatic framework to consider the findings of this systematic review. These criteria are not definitive rules, rather they provide an analytical framework to consider whether cause and effect is the reasonable inference [129]. The weight of evidence in this systematic review supports a causative relationship between crowding and promotion of GAS transmission, and its rheumatic sequelae. Particular strengths were that evidence was collected across many study types including prospective and retrospective cohort studies and covered diverse population groups globally; features that enhance confidence in casual interpretation [129]. While the presence of a biological gradient may not rule out confounding (as a confounder may also exert a dose-response effect), it provides compelling evidence of a causative nature of this association. Furthermore, there is firm biologic plausibility, since GAS infection is transmitted by close contact and the respiratory route [130]. It can be inferred that crowding plays a critical mediating role between poverty and RHD prevalence, supported by those studies which included multivariable analyses [35, 40, 74, 90, 97, 105].

Overall socioeconomic status also effectively met the Bradford Hill criteria for causation of the outcomes of interest. Since this overarching category is collinear with (and either determined by or a determinant of) the other measures examined, it is not possible to tease out specifics relating to how each criterion was met. In general, it is well understood that rising socioeconomic status, prior to availability of penicillin, was associated with a steady decline in death rates from ARF in industrialised nations [131]. It is likely the combination of these adverse factors that creates the environment that drives ARF and RHD risk among socioeconomically disadvantaged populations. This compounding effect also explains why these factors individually do not necessarily exert the same risk.

The other factors explored in this review had insufficient evidence to suggest causal links. Dwelling characteristics frequently displayed an association with GAS infection, ARF, or RHD risk, but measures were so heterogeneous and context specific that generalizability is impossible. Further, authors consistently omitted a proposed mechanism to explain their findings. The role of fomites in GAS transmission was not supported [106, 108]. Soap and hand-washing had an impressive effect on reducing impetigo, but only one study explored this intervention [107]. Findings relating to education and employment were inconsistent. Specific nutritional interventions were suggested and tested, but lacked scale and consistency. Studies that demonstrated the association of low income and risk of GAS infection and associated diseases did not explore which economic deficits confer the risk (e.g. unaffordability of health care, poor diet etc.). General explorations of income as a social determinant of heath cite that low income exerts a risk to health through material deprivation (medical care, nutrition, housing, and sanitation) and social participation (education, employment) [120]. The association of income in this instance with RHD and its antecedents is likely a consequence of these intermediary factors.

### Strengths and limitations

This systematic review is the most extensive review to date targeting observational and experimental studies in the area of social determinants of health and GAS infection, ARF and RHD. A limitation was the exclusion of non-English articles; nevertheless, a wide variety of countries were represented. A further limitation was that all stages of article appraisal were undertaken by one reviewer. However, a strict methodological process was followed utilising assessment tools designed for each study type allowing greater specificity in appraisal and uniformity in the reviewing process.

The poor quality of this body of evidence is the most critical limitation in guiding preventative actions; however, it would be erroneous to reject all findings. Rather it should be considered how probable or not it is that this diverse collection of studies dispersed in time, person and place could all be flawed and biased in the same way. Confounding is another important consideration in this review. Simple cause-effect relationships within social determinants of health are not readily apparent [13, 132]- rather they are complex and are characterized by multiple determinants, multiple outcomes, and multiple potential interactions [133]. That few included studies undertook multivariate analysis is a limitation of this review, which is why it was important to highlight their results explicitly in this analysis.

Many studies presented outcomes at a group or ecological level rather than an individual level. However, assessing social determinants of health at the group level is also important because people do not live in isolation; a population level approach ensures that a wide variety of contributing settings and activities are not inadvertently overlooked [134]. Also, these studies designs can reflect the interventions addressing the social determinants of health that are aimed at the population level.

Finally, reporting bias is an important consideration in systematic reviews. The factors extracted for this review were frequently not the primary outcome of studies, and so their positive, neutral or negative findings would be less likely to influence whether a paper was published.

### Implications for ARF and RHD control

Rather than re-describe the problem, the aim of this research was to guide solutions. In ARF and RHD where treatment is logistically intensive and painful, [135] and vaccines or mass drug administration are not currently available options, the case for action on the social determinants that drive ARF and RHD risk is unquestionably convincing. Several candidate vaccines are in development, [136] but even if found to be safe and effective, not all at-risk populations would be able to readily access a new vaccine.

Based on these findings, we recommend that ARF and RHD control programs should address household crowding—particularly in high-income countries where funding and resourcing is more feasible. Structural crowding (inadequate living space including number of bedrooms) must be addressed in collaboration with designers and providers of public housing in partnerships that recognise housing needs to support good health.

Functional crowding (people sharing living spaces for safety, warmth or social cohesion, especially in traditional societies) is more difficult to address, requiring in-depth cultural understanding. For example, in Australian Aboriginal societies, rights and obligations around accommodating extra people in a house must be respected in interventions to reduce household crowding [137]. Where close living is important culturally, ways to live safely in larger households, focusing on ensuring adequate health literacy and washing facilities, need to be implemented.

Site-specific tailoring of interventions are needed: publically-funded interventions in a cold climate high RHD-burden setting for instance include the provision of household insulation and heating to reduce functional bedroom crowding [138]. Conversely, in hot climates, constructing community swimming pools is an effective intervention to decrease GAS skin infections, [139] though pools must be adequately managed so as to not introduce other health risks. Further practical interventions to mitigate the health risks arising from crowding include: community development projects to improve health literacy pertaining to infection transmission; creating community demand for sanitation and hygiene; [140] and effective community consultation about factors to motivate change in behaviour.

Interventions to tackle socioeconomic status may be considered beyond the reach of medical research and health service delivery, but this is not so. Research and service delivery initiatives in high RHD-burden settings should ensure that they provide opportunities for community engagement and employment; and funding bodies should ensure that initiatives supporting strengthening of socioeconomic status are valued as being critical in disease prevention.

RHD control programs, where they exist, should aim to routinely collect objective metrics on social and environmental factors to further inform advocacy, tailor it to local needs and add to the evidence base. Outcomes of advocacy should be interventions targeting social determinants such as crowding reduction and hygiene hardware improvement; and these should be accompanied by rigorous evaluation and sharing of findings.

### Conclusion

Findings from this systematic review will be able to inform guidelines and policies regarding primordial-level preventative interventions against GAS infection, ARF and RHD. The wide body of evidence exploring links between certain social and environmental factors and these conditions is limited by poor quality and a lack of interventional studies. Historically, this has hampered the ability of control programs and guidelines to legitimately target these factors. However, when scrutinized against the Bradford Hill criteria assessing the evidence of a causal relationship, the link between overall socioeconomic status and crowding with ARF and RHD risk can be satisfactorily viewed as one of cause and effect. This clear and powerful message should be reflected in ARF and RHD control efforts. A critical role for ARF and RHD control programs and registers is to routinely collect and analyse data on these social determinants alongside clinical markers of case management to inform future advocacy and interventions.

## Supporting information

S1 Checklist PRISMA checklist. (DOCX)

S1 Text National Institute of health Study Quality Assessment Tool template. (PDF)

S1 Fig PRISMA flow diagram. (TIF)

S1 Table Search strategy. (PDF)

S2 Table Summary of crowding and GAS infection, ARF and RHD. (DOCX)

S3 Table Summary of dwelling characteristics and facilities and GAS infection, ARF and RHD. (DOCX)

S4 Table Summary of education and GAS infection, ARF and RHD. (DOCX)

S5 Table Summary of employment and GAS infection, ARF and RHD. (DOCX)

S6 Table Summary of income and GAS infection, ARF and RHD. (DOCX)

S7 Table Summary of nutrition and GAS infection, ARF and RHD. (DOCX)

S8 Table Summary of socioeconomic status and GAS infection, ARF and RHD. (DOCX)
