# Supplementary material for: The role of social determinants of health in the risk and prevention of group A streptococcal infection, acute rheumatic fever and rheumatic heart disease: A systematic review
Source: PLoS Negl Trop Dis. 2018 Jun 13;12(6):e0006577. doi: 10.1371/journal.pntd.0006577 (PMC6016946; doi:10.1371/journal.pntd.0006577)
Supplement: S1 Text — (PDF) [file pntd.0006577.s002.pdf]

S1 Supporting Information. National Institute of Health Study Quality Assessment Tool

Quality Assessment of Case-Control Studies

Criteria	Comments	Yes	No	Other (CD, NR, NA)*
1. Was the research question or objective in this paper clearly stated and appropriate?				
2. Was the study population clearly specified and defined?				
3. Did the authors include a sample size justification?				
4. Were controls selected or recruited from the same or similar population that gave rise to the cases (including the same timeframe)?				
5. Were the definitions, inclusion and exclusion criteria, algorithms or processes used to identify or select cases and controls valid, reliable, and implemented consistently across all study participants?				
6. Were the cases clearly defined and differentiated from controls?				
7. If less than 100 percent of eligible cases and/or controls were selected for the study, were the cases and/or controls randomly selected from those eligible?				
8. Was there use of concurrent controls?				
9. Were the investigators able to confirm that the exposure/risk occurred prior to the development of the condition or event that defined a participant as a case?				

10. Were the measures of exposure/risk clearly defined, valid, reliable, and implemented consistently (including the same time period) across all study participants?				
11. Were the assessors of exposure/risk blinded to the case or control status of participants?				
12. Were key potential confounding variables measured and adjusted statistically in the analyses? If matching was used, did the investigators account for matching during study analysis?				

**Quality Rating (Good, Fair, or Poor) (see guidance)**
Rater #1 initials:
Rater #2 initials:
Additional Comments (If POOR, please state why):

*CD, cannot determine; NA, not applicable; NR, not reported

Quality Assessment Tool for Case Series Studies

Criteria	Coments	Yes	No	Other (CD, NR, NA)*
1. Was the study question or objective clearly stated?				
2. Was the study population clearly and fully described, including a case definition?				
3. Were the cases consecutive?				
4. Were the subjects comparable?				
5. Was the intervention clearly described?				
6. Were the outcome measures clearly defined, valid, reliable, and implemented consistently across all study participants?				
7. Was the length of follow-up adequate?				
8. Were the statistical methods well-described?				
9. Were the results well-described?				

**Quality Rating (Good, Fair, or Poor)**
Rater #1 initials:
Rater #2 initials:
Additional Comments (If POOR, please state why):

*CD, cannot determine; NA, not applicable; NR, not reported

Quality Assessment of Controlled Intervention Studies

Criteria	Comments	Yes	No	Other (CD, NR, NA)*
1. Was the study described as randomized, a randomized trial, a randomized clinical trial, or an RCT?				
2. Was the method of randomization adequate (i.e., use of randomly generated assignment)?				
3. Was the treatment allocation concealed (so that assignments could not be predicted)?				
4. Were study participants and providers blinded to treatment group assignment?				
5. Were the people assessing the outcomes blinded to the participants' group assignments?				
6. Were the groups similar at baseline on important characteristics that could affect outcomes (e.g., demographics, risk factors, co-morbid conditions)?				
7. Was the overall drop-out rate from the study at endpoint 20% or lower of the number allocated to treatment?				
8. Was the differential drop-out rate (between treatment groups) at endpoint 15 percentage points or lower?				
9. Was there high adherence to the intervention protocols for each treatment group?				
10. Were other interventions avoided or similar in the groups (e.g., similar background treatments)?				
11. Were outcomes assessed using valid and reliable measures, implemented consistently across all study				

participants?				
12. Did the authors report that the sample size was sufficiently large to be able to detect a difference in the main outcome between groups with at least 80% power?				
13. Were outcomes reported or subgroups analyzed prespecified (i.e., identified before analyses were conducted)?				
14. Were all randomized participants analyzed in the group to which they were originally assigned, i.e., did they use an intention-to-treat analysis?				

**Quality Rating (Good, Fair, or Poor) (see guidance)**
Rater #1 initials:
Rater #2 initials:
Additional Comments (If POOR, please state why):

*CD, cannot determine; NA, not applicable; NR, not reported

Quality Assessment Tool for Observational Cohort and Cross-Sectional Studies

Criteria	Comment	Yes	No	Other (CD, NR, NA)*
1. Was the research question or objective in this paper clearly stated?				
2. Was the study population clearly specified and defined?				
3. Was the participation rate of eligible persons at least 50%?				
4. Were all the subjects selected or recruited from the same or similar populations (including the same time period)? Were inclusion and exclusion criteria for being in the study prespecified and applied uniformly to all participants?				
5. Was a sample size justification, power description, or variance and effect estimates provided?				
6. For the analyses in this paper, were the exposure(s) of interest measured prior to the outcome(s) being measured?				
7. Was the timeframe sufficient so that one could reasonably expect to see an association between exposure and outcome if it existed?				
8. For exposures that can vary in amount or level, did the study examine different levels of the exposure as related to the outcome (e.g., categories of exposure, or exposure measured as continuous variable)?				
9. Were the exposure measures (independent variables) clearly defined, valid, reliable, and implemented consistently across all study participants?				
10. Was the exposure(s) assessed more than once over time?				
11. Were the outcome measures (dependent variables) clearly defined, valid, reliable, and implemented consistently				

across all study participants?				
12. Were the outcome assessors blinded to the exposure status of participants?				
13. Was loss to follow-up after baseline 20% or less?				
14. Were key potential confounding variables measured and adjusted statistically for their impact on the relationship between exposure(s) and outcome(s)?				

**Quality Rating (Good, Fair, or Poor) (see guidance)**
Rater #1 initials:
Rater #2 initials:
Additional Comments (If POOR, please state why):

*CD, cannot determine; NA, not applicable; NR, not reported

Quality Assessment Tool for Observational Cohort and Cross-Sectional Studies

Criteria	Comment	Yes	No	Other (CD, NR, NA)*
1. Was the research question or objective in this paper clearly stated?				
2. Was the study population clearly specified and defined?				
3. Were the eligibility of the ecologic units, as well as their sources and any sampling methods used given? Did the authors provide a rationale for their selection? If the units were aggregated for the study, was it explained how this was done and the criteria by which they were constructed?				
4. Were all the subjects selected or recruited from the same or similar populations (including the same time period)? Were inclusion and exclusion criteria for being in the study prespecified and applied uniformly to all participants?				
5. Was a sample size justification, power description, or variance and effect estimates provided?				
6. For the analyses in this paper, were the exposure(s) of interest measured prior to the outcome(s) being measured?				
7. Was the timeframe sufficient so that one could reasonably expect to see an association between exposure and outcome if it existed?				
8. For exposures that can vary in amount or level, did the study examine different levels of the exposure as related to the outcome (e.g., categories of exposure, or exposure measured as continuous variable)?				
9. Were the exposure measures (independent variables) clearly defined, valid, reliable, and implemented consistently across all study participants?	.			

10. Was the exposure(s) assessed more than once over time?				
11. Were the outcome measures (dependent variables) clearly defined, valid, reliable, and implemented consistently across all study participants?				
12. Were the outcome assessors blinded to the exposure status of participants?				
13. Was loss to follow-up after baseline 20% or less?				
14. Were key potential confounding variables measured and adjusted statistically for their impact on the relationship between exposure(s) and outcome(s)?				

**Quality Rating (Good, Fair, or Poor) (see guidance)**
Rater #1 initials:
Rater #2 initials:
Additional Comments (If POOR, please state why):

*CD, cannot determine; NA, not applicable; NR, not reported
