# Supplementary material for: The role of social determinants of health in the risk and prevention of group A streptococcal infection, acute rheumatic fever and rheumatic heart disease: A systematic review
Source: PLoS Negl Trop Dis. 2018 Jun 13;12(6):e0006577. doi: 10.1371/journal.pntd.0006577 (PMC6016946; doi:10.1371/journal.pntd.0006577)
Supplement: S1 Table — (PDF) [file pntd.0006577.s004.pdf]

Supplementary Table 1: Search strategy for "The role of social determinants of health in the risk and prevention of group A streptococcal infection, acute rheumatic fever and rheumatic heart disease: A systematic review"

Database	Search strategy
Pubmed (MeSH Terms)	acute rheumatic fever[MeSH Terms] OR rheumatic heart disease[MeSH Terms] OR streptococcus pyogenes[MeSH Terms]<br><br>AND<br>housing[MeSH Terms] OR environment and public health[MeSH Terms] OR infectious disease transmission[MeSH Terms] OR social environment[MeSH Terms] OR poverty[MeSH Terms] OR socioeconomic fact ORs[MeSH Terms] OR residence characteristics[MeSH Terms] OR social conditions[MeSH Terms] OR sanitation[MeSH Terms] OR water supply[MeSH Terms] OR life style[MeSH Terms] OR poverty areas<br><br>AND<br><br>Intervention study[MeSH Terms] OR health promotion[MeSH Terms] OR epidemiology[MeSH Terms] OR population surveillance[MeSH Terms] OR primary prevention[MeSH Terms] OR health education[MeSH Terms] OR community survey[MeSH Terms]<br><br>WITH LIMITS<br>Randomized Controlled Trial; Clinical Trial; Controlled Clinical Trial; Evaluation Studies; Journal Article; Meta-Analysis; Multicenter Study; Observational Study; Systematic Reviews; Abstract; Humans; English
Pubmed (Key word search)	Acute rheumatic fever OR Rheumatic heart disease OR Group A streptococcus<br><br>AND<br>housing OR environmental factors OR socioeconomic OR poverty OR crowding OR overcrowding OR nutrition<br><br>WITH LIMITS:<br>Humans; English
Cochrane library (Key word search)	Acute rheumatic fever OR Rheumatic heart disease OR Group A streptococcus<br><br>AND<br>housing OR environmental factors OR socioeconomic OR poverty OR crowding OR overcrowding OR nutrition<br><br>WITH LIMITS:<br>Humans; English
Embase (Emtree term search)	'rheumatic fever'/exp OR 'rheumatic fever' OR 'rheumatic heart disease'/exp OR 'rheumatic heart disease' OR 'streptococcus group a'/exp OR 'streptococcus group a'<br><br>AND<br><br>'demography'/exp OR 'demography' OR 'disease transmission'/exp OR 'disease transmission' OR 'social environment'/exp OR 'social environment' OR 'poverty'/exp

	OR 'poverty' OR 'socioeconomics'/exp OR 'socioeconomics' OR 'social status'/exp OR 'social status' OR 'sanitation'/exp OR 'sanitation' OR 'water supply'/exp OR 'water supply' OR 'lifestyle'/exp OR 'lifestyle'<br><br>AND<br>'intervention study'/exp OR 'intervention study' OR 'health promotion'/exp OR 'health promotion'<br>OR 'epidemiology'/exp OR 'epidemiology' OR 'health survey'/exp OR 'health survey' OR 'health education'/exp OR 'health education' OR 'community surveys'<br><br>WITH LIMITS:<br>Humans; English; Articles
