# Supplementary material for: The role of social determinants of health in the risk and prevention of group A streptococcal infection, acute rheumatic fever and rheumatic heart disease: A systematic review
Source: PLoS Negl Trop Dis. 2018 Jun 13;12(6):e0006577. doi: 10.1371/journal.pntd.0006577 (PMC6016946; doi:10.1371/journal.pntd.0006577)
Supplement: S2 Table — (DOCX) [file pntd.0006577.s005.docx]

S2 Table. Summary of crowding and GAS infection, ARF and RHD

| Study details | Aim of study | Study design | Study population and setting | Measure of crowding and strata and methods | Measure of outcome (GAS, ARF, RHD) | Outcome incidence/ prevalence | Results: univariate | Results: Multivariate | Study quality |
| --- | --- | --- | --- | --- | --- | --- | --- | --- | --- |
| Adanja et al 1988 | To test the hypothesis regarding the influence of socioeconomic and some other factors on the occurrence of ARF. | Case control | 148 with first ARF attack, 444 controls matched for age, sex and place of residence (1:3)  Serbia | Number of persons in the home (2-4, 5-6, 7+)  Number of children in a household (0-1, 2, ≥3)  ≥2 persons per room  ≥2 persons per bedroom  ≥2 persons per bed  <5m^2^ Living space per capita | ARF using revised Jones criteria | NA | **Positive association** Living space <5m^2^ 8.1% vs. 3.1% RR 2.83 (p=0.018)  ≥2 persons per room 86.5% vs. 81.1% RR 1.72 (p=0.021)  ≥2 per bed 49.3% vs. 40.5% RR 1.65 (p=0.040)  **No association**  Number of persons in a household  Number of children in a household  Number of persons per bedroom. |  | Poor to fair: cannot determine temporal association of exposure and outcome; no multivariate analysis. |
| Dobson et al 2012 | To investigate the role of environmental factors for RHD in Fiji. | Case control | 80 children aged 5-15 years with RHD and 80 age and sex matched controls  Fiji | Mean people in the home  Mean people per bedroom  Mean children per classroom | Definite RHD diagnosed on echocardiogram using WHO criteria | NA | **No association**  Mean people in the home  Mean people per bedrooms  Mean children per classroom |  | Poor: no power calculations, unstated number of controls from different source, participation rate 61%. |
| Grave 1957 | To investigate the factors of social and emotional forces in the aetiology of rheumatic fever. | Case control | 122 children aged between 2 and 12 years with ARF, 100 controls from outpatient clinics within same age range  Sydney, Australia | < 300 cubic feet of sleeping space per person  ≥4 children in household | ARF diagnosed on criteria of the Rheumatic fever council of the American Heart Association | NA | **Possible association** <300 feet^3^ 31% vs. 19%  ≥4 children 44% vs. 27%  (no test of significance) |  | Poor: unmatched controls and no adjusting for differences, no power calculations, no test of significance |
| Gray et al 1952 | To follow up a previous study with a long period of observation to compare rheumatic and control families with respect to hereditary and certain environmental factors which might be responsible for the familial prevalence of rheumatic fever. | Case control | 40 families with a ARF case, 30 control families with a scarlet fever case  Connecticut, USA | >1 persons per room  >1 persons per bedroom  >2 persons per bed | ARF diagnosed by physicians at time of acute episode (criteria not reported).  RHD diagnosed using clinical criteria of the New York and American Heart Association | NA | **Possible association**  Measured in 1930-1931  >1 persons per room 85.9% vs. 76.6%  >1 persons per bedroom 59.8% vs.26.3%  >2 persons per bed 23.4 vs. 6.7%  (no test of significance)  **No association**  Measured in 1948-1949  >1 persons per room  >1 persons per bedroom  >2 persons per bed |  | Poor: high proportion of cases lost to follow up, test of significance not reported |
| Hewitt & Stewart 1952 | This study deals with the social background of acute rheumatism. | Case control | 793 children aged 5-14 years  Sheffield, UK | ≥6 persons per household  Persons per room  Overcrowding Y/N (not defined).  People per bedrooms of cases  Household size | Acute rheumatism diagnosis based on notification criteria of the County Borough | NA | **Positive association** ‘Significantly’ larger proportion of cases living in households of ≥6 persons  ‘Significantly’ smaller proportion of cases in households with ≥6 rooms  Household size had continuous effect on morbidity ratio  (statistical test not reported)  **No association** Overcrowding  People per bedrooms of cases |  | Poor: no baseline comparison, numbers not reported, participation rate not reported. |
| Riaz et al 2013 | To identify the risk factors of ARF and to explore the risk factors of RHD among ARF patients | Case control | 103 RHD cases, 103 ARF cases, 207 controls  Bangladesh | Family size >4  Number of siblings >2  People per room  Family size | ARF diagnosed using modified Jones criteria  RHD diagnosed by doppler echocardiography | NA | **Positive association**  For RHD:  >2 siblings 50.0% non-ARF vs. 68.0% RHD OR 2.1 (1.3-3.4)  >3 persons per room 52.2% non-ARF vs. 65.0% RHD OR 1.7 (1.1-2.7)  **No association**  For ARF:  >2 siblings  >3 persons per room  **Negative association**  For RHD:  Family size >4 OR 0.54 (0.3-0.9)  For ARF:  Family size >4 OR 0.4 (0.26-0.7) | **Positive association**  For ARF:  >2 siblings OR 3.1 (1.5-6.3)  For RHD:  Number of siblings >2 OR 4.4 (2.2-8.7)  >3 per room OR 1.9 (1.0-3.4)  For RHD in ARF patients:  >3 per room OR 2.4 (1.2-4.7)  **No association**  People per room >3 for ARF  Number of siblings >2 for RHD in ARF case  **Negative association**  Family size >5 OR 0.3 (0.2-0.7) for ARF and OR 0.5 (0.2-0.9) for RHD | Fair: no matching, blinding of assessors not stated. |
| Kurahara et al 2006 | To determine factors in prevalence rates of ARF in a multiethnic population. | Case control | 26 cases ARF, 41 controls with other heart condition (all on Medicaid)  Hawaii, USA | Household size Number of children per house  Number per subject’s bedroom | ARF diagnosed using modified Jones criteria | NA | **Positive association**  Mean household size 7.3 vs 5.5 (p=0.01)  Number children per house 4.1 vs. 3.0 (p=0.03)  **No association**  Children in subject’s bedroom |  | Fair: no matching, small sample size |
| Levine et al 1966 | A follow up study to define the extent of interfamilial spread of streptococcus within a population of servicemen and their families | Case control | 1, 065 GAS cases, 709 GAS negative cases  Army base, USA | Number per family | Throat swab culture | NA | **Positive association**  Significant correlation between number per family and proportion of GAS positive swabs   - 2: 25% - 3: 22% - 4: 28% - 5: 33% - 6: 38% - 7: 35% - 8: 37% - 9: 42% - >9: 36%   (statistical test not reported) |  | Poor to fair: no matching or comparison of baseline characteristics, limited variables measured. |
| Okello et al 2012 | To investigate the role of socioeconomic and environmental factors in the pathogenesis of RHD in Ugandan patients. | Case control | 243 RHD cases, 243 controls aged 5- 60 years  Uganda | People per house >8  Space per person < 90 feet^2^ | RHD diagnosed using history ARF, clinical examination, echocardiogram criteria | NA | **Positive association**  People per house >8 OR 1.98 (1.4-2.5)  Space per person < 90 feet^2^ OR 8.3 (6.1-10.4) | **Positive association**  Space per person < 90 square feet OR 1.35 (1.1-1.56) | Fair: unmatched controls, but randomly chosen and multivariate analysis performed, time period of participant selection not stated. |
| Vlajinac et al 1991 | To investigate the independent, unconfounded effect of risk factors for ARF identified in a previous study conducted on this population. | Case control | 148 with first ARF attack, 444 controls matched for age, sex and place of residence (1:3)  Serbia | Space per person < 5m^2^  >2 people per room  Sleeping in bed with another person | ARF diagnosed using revised Jones criteria | NA |  | **No association**  Space per person < 5m^2^  >2 people per room  Sleeping in bed with another person | Fair: temporal association of exposure and outcome not explicitly stated; |
| Vlajinac et al 1989 | To test the hypothesis that socio-economic factors are related to rheumatic fever and make an additional comparison for those with and without a history of sore throat. | Case control | 148 with first ARF attack, 444 controls matched for age, sex and place of residence (1:3)  Serbia | >2 people per room  Sleeping in bed with another person | ARF diagnosed using revised Jones criteria  Frequent sore throat= >1/year | NA | **Positive association**  Participants without frequent sore throat:  >2 people per room RR 2.31 (1.38-3.90)  Sleeping in bed with another person RR 2.11 (1.26-3.55)  >2 people per room and sleeping in bed with another person for participants RR 3.57 (1.71-7.51)  **No association**  With frequent sore throat:  >2people per room  Sleeping in bed with another person |  | Poor to fair: temporal association of exposure and outcome not explicitly stated; stratified only by sore throat frequency. |
| Zaman et al 1997 | To examine data on patients with proven GAS infection presenting to a ARF hospital to identify socio-economic factors that may need further exploration. | Case control | 44 ARF cases, 86 controls aged 5-20 years with recent GAS infection  Dhaka, Bangladesh | Mean family size  Dwelling space  Family size >6  Dwelling space per person <75 feet^2^ | ARF diagnosed on revised Jones criteria | NA | **Positive association**  Mean family size 6 vs. 5 (p=0.04)  Dwelling space square feet per person 83 vs. 138 (p<0.01)  **No association**  Family size >6  Dwelling space per person <75 feet^2^ | **No association** Family size >6 | Fair to good: no power calculations |
| Zaman et al 1998 | To explore further the nutritional factors that may be associated with ARF. | Case control | 60 ARF cases, 104 controls aged 5-20 years with recent GAS infection  Dhaka, Bangladesh | Number of siblings  Family size  Persons sharing a bedroom with subject  Persons per dwelling room | ARF diagnosed using updated Jones criteria | NA | **Positive association**  Number of siblings 4.4 vs 3.4 (p=0.001)  **No association**  Family size  Persons sharing bedroom with subject  Persons per dwelling room |  | Fair to good: no power calculations. |
| Bach et al 1996 | To report how the Martinique/Guadeloupe ARF eradication programme was set up and its results over 10 years | Case series | Not stated  French Caribbean | >4 children  Average number of children per family | ARF diagnosed using modified Jones criteria | 19.6/ 100,000 residents <20 years | **Positive association**  Number of children >4 53% Martinique families vs. 5% general population (p<0.001)  Mean number of children 5.6 vs. 3.7 (p<0.01) |  | Poor: limited analysis and incomplete reporting of figures |
| Lennon et al 1988 | To explore the possible role of impetigo in rheumatic fever in areas where impetigo and rheumatic fever are common. | Case series | 240 definite ARF cases  Auckland, New Zealand | Household size | Definite ARF diagnosed using revised Jones criteria. Probable case if met modified Jones criteria | 22/100,000 for children < 15 years | **Possible association**  Persons per household 5.6 vs. 3.0 (national mean)  (no test of significance) |  | Poor: poor statistical methods regarding socio-environmental factors |
| Wannamaker 1954 | To review pertinent reports of other authors and some of the findings of the Streptococcal Disease Laboratory related to infections of the upper respiratory tract by group A streptococci. | Case series | Not stated  Warren Air Force Base, USA | Distance between beds of GAS carriers and new cases 0-10 feet, 11-20 feet, >20 feet.  Number of carriers in a sleeping room | Throat and nasal swab culture and typing | NA | **Possible association** Higher acquisition rate correlated to bed distance between carriers and new cases (graph only, no test of significance)  Rate of acquisition of GAS correlated to number of carriers in barrack group (graph only, no test of significance) |  | Poor: no figures or methods presented. |
| Westlake et al 1990 | To assess the current incidence of ARF, to characterise its epidemiology, to assess the antecedent symptoms and treatment of such and to describe the clinical manifestations of ARF in these patients. | Case series | 26 cases ARF aged 4-14 years  Tennessee, USA | Family size  Persons per bedroom | ARF diagnosed using revised Jones criteria |  | **Possible association**  Mean family size 4.6 cases vs. 3.2 general population  Mean persons per bedroom 1.6 cases vs.0.7 general population  (no test of significance) |  | Poor: no statistical tests undertaken, small sample. |
| Breese et al 1955 | To find out how often a streptococcal infection is communicated from a sick child to other members of the family and what factors may influence the communicability under the conditions of private paediatric practice. | Cohort | 363 families with 428 primary GAS cases  New York state, USA | Number of children per family | Throat swab culture for GAS |  | **No association**  Number of children per family |  | Poor: poor statistics, attrition not reported. |
| Coggon et al 1993 | To examine the associations with overall mortality and specific diseases (RHD) that might be expected to relate to specific aspects of housing. | Cohort | 51 deaths from RHD between 1951-1989 | Crowding index= total persons resident in 1939/persons allowed (by housing regulations) in 1936  Persons per bedrooms= total persons resident in 1939/number of bedrooms 1936 | Cause of death listed as RHD on Death Certificate |  | **No association**  RHD mortality between 1951-1989 and crowding index, persons per bedrooms |  | Poor: unreliable measures of exposures and outcome, sampling method not reported. |
| Eriksson et al 2013 | To examine the association between maternal size, neonatal body size, placental size and the occurrence of chronic rheumatic heart disease within the Helsinki Birth Cohort | Cohort | 101 RHD cases  Helsinki, Finland | Number of people per household: ≤3,4, 5, ≥6  Number of people per room | RHD cases ascertained from centralised national hospital admission and death database using ICD codes |  | **Positive association** Number of people per household:   - ≤3 (reference) - 4 HR 2.1 (1.1-4.0) - 5 HR 2.1 (1.1-4.2) - ≥6 HR 2.1 (1.0-4.3)   **No association**  Number of people per room |  | Fair: no confounders/no multivariate analysis, no power calculation. |
| McDonald et al 2006 | To conduct prospective surveillance for streptococcal infection and colonization of the throat and skin among families of people with known previous cases of ARF and/or RHD in remote Aboriginal communities to further elucidate the potential relationship between pyoderma and ARF. To document the burden of GAS pharyngitis and pyoderma, age distribution, seasonal variation, and the link to domestic overcrowding. | Cohort (enrolment of household, not individuals) | 1173 adults and children from 49 households  3 remote communities, Australia | People per bedroom | Throat swab culture | Median point prevalence GAS positive throat swab 3.7% | **Positive association**  No. cases of pyoderma per household consultation and no. of people per bedroom in community 1 (r^2^ =0.62)  **No association**  Household crowding and GAS, βHS rates in throat. |  | Fair: turnover and attrition of individual participants. |
| McDonald et al 2008 | To collect throat swabs and pyoderma swabs from children in households at high risk for ARF to examine the household and community epidemiology of pharyngitis and pyoderma and determine whether there were features unique to the study population including household and personal acquisition, length of carriage, secondary transmission and the effect of age. | Cohort (enrolment by household, not individuals) | 1,173 individuals from 49 households with history of either an ARF or RHD case  3 remote communities, Australia | People per house | Throat and wound swab GAS culture and *emm*ST typing | >25/1000 all ages | **Positive association** Correlation between *emm*ST acquisition per household year and household size for community 1 Pearson coefficient =0.88 (p<0.001); and for community 3 0.68 (p<0.001) |  | Fair: turnover of individual participants within households. |
| Mirabel et al 2015 | To address the outcomes and modalities of RHD screening through a cohort of children with and without RHD who took part in the first large RHD echocardiography based surveillance programme. | Cohort | 114 cases of RHD from cohort of 157 and 227 controls selected randomly from classmates, matched for ethnicity and classroom  New Caledonia | Mean number of people in house  ≥3 persons per bedroom  Number of siblings by mother :≤2, 3-5, ≥6 | Persistence of RHD diagnosed using World Heart Foundation criteria.    ARF diagnosed using Australian Guideline for Prevention, Diagnosis and Management of ARF and RHD. | RHD: 890/100,000  ARF:  10.28/1000/year | **Positive association**  ≥3 per bedroom 35.6% Persistent RHD vs. 8.3% normal echo (p=0.003)  Number of people in house 6 RHD vs. 5 non-RHD  ≥3 per bedroom 30.1% RHD vs.27.4% non-RHD (no test of significance)  **No association**  Number of siblings | **Positive association**  Persistent RHD vs. normal echo  ≥3 per bedroom OR 8.27 (1.67-41.08) | Fair: no power calculation, >20% lost to follow up. |
| Nandi et al 2001 | To estimate the incidence and risk factors for GAS sore throat among school-aged children in northern India | Cohort | 536 children aged 5-15 years in 26 peri-urban slum households  Chandigarh, India | Area per person in house m^2^ :<2.32, 2.32 to <3.09, 3.1 to <3.72, >3.72 | Throat swab culture for GAS | 1 episode/child/year among 5-15 year olds. | **No association**  Area per person in house |  | Poor to fair: no power calculations, no multivariate analysis. |
| Ransome et al 1983 | To investigate the carrier rate of Lancefield GAβHS in two cohorts of school children, one Coloured and one Indian, and the influence of the degree of crowding in their homes. | Cohort | 120 Coloured children, 126 Indian children  Johannesburg, South Africa | Persons per bedroom | Throat swab culture for GAS | NA | **No association**  Persons per bedroom |  | Poor: limited methodology reported, no power calculations. |
| Tay et al 1981 | To collect information pertaining to the following points: the carriership of β-haemolytic streptococcal throat and skin diseases; the incidence of various serological groups of haemolytic streptococci and of the types of GAS; the role played by the various factors like race, season of the year, socio-economic status etc. on the clinical, epidemiological and microbiological patterns of the streptococcal carriership and of the disease; the assessment of the best therapy regime for streptococcal disease applicable in the local conditions. | Cohort | 491 primary school students  Singapore | Persons per room: ≤2, >2- ≤4, >4 | Throat and skin swab cultures | Overall cumulative incidence of GAS throat carrier rate was 46.7% | **Positive association**  Persons per room & GAS incidence:   - ≤2 23.7% - >2-≤4 54.5% - >4 58.1%   (for ≤2 vs. >2 p<0.05) |  | Poor to fair: >20% attrition, no power calculation, poor reporting of statistics. |
| Bernstein 1957 | To report a study of the comparative incidence and spread of common respiratory disease among recruits who live in closed bay (compartmentalised) or open bay (dormitory style) barracks during training at Sampson Air Force Base, to compare these two types of housing to ascertain their relative desirabilities with respect to their hindrance of the spread of GAS infections, influenza, and acute respiratory disease of viral etiology. | Control trial | 15, 090 male Air Force recruits  Sampson Air Base,  USA. | Closed bay (compartmentalised) or open bay (dormitory style) barracks | Throat culture for GAS. | NA | **No association**  Style of barracks |  | Poor: no baseline comparison, not randomised. |
| Ba-Siddik et al 2011 | To estimate the prevalence of RHD among school-children aged 5-16 years in Aden (Yemen). | Cross section | 6,000 school children aged 5-16 years  Aden, Yemen | >4 persons per bedroom | Definitive RHD diagnosed using modified Duckett Jones’ criteria and WHO criteria for Doppler abnormalities, in those with clinical murmurs. | 36.5/1000 children | **Positive association**  >4 per bedroom 64.8% RHD vs. 37.1% non RHD (χ^2^ p<0.001) |  | Poor to fair: no adjustment, non-blinded assessors |
| Faruq et al 1995 | To detect the status of βHS infection in the throat of children and the role of environmental factors in the infection. | Cross section | 601 children aged 5-15 years  Dhaka, Bangladesh | Large family= > 6 members | Throat culture for βHS, GAS | βHS 22%  GAS 4.2% | **Positive association**  GAS+ prevalence 8.2% large family vs. 2.7% small family (p<0.01) |  | Poor: non-participation not reported, no multivariate analysis. |
| Hammon et al 1950 | A preliminary study to explore the usefulness of a test to indicate past infection with poliomyelitis and the disease’s possible correlation to streptococcal infections. | Cross section | 653 school children aged 1-15 years  California, USA | Number of children per family: 1-2, ≥3 | ASOT |  | **Positive association**  ASOT+ children per family sized 1-2 vs. ≥3 in stratified by high/low socioeconomic group:  Population 1  High 42% 1-2 vs. 49% (χ^2^ 1.29)  Low 46% vs. 59% (χ^2^ 2.88)  Population 2  High 33% vs. 46% (χ^2^ 1.97)  Low 39% vs. 69% (χ^2^ 8.07)  (Total of χ^2^14.21, p<0.01) |  | Poor: inadequate description of methods. |
| Likitnukal et al 1994 | To evaluate the factors influencing streptococci colonization of school age children. | Cross section | 1,547 school children aged 6-11 years  Bangkok, Thailand | >5 persons in household  ≥3 children in household  >3 people sharing a bedroom | Throat swab culture for βHS and GAS | GAS 18%  βHS 47% | **No association**  >5 in household  ≥3 children in household  >3 people sharing a bedroom |  | Poor: high attrition, no power calculation. |
| Longo-Mbenza et al 1998 | A study of the prevalence of RHD in children of Kinshasa using echocardiography to confirm the diagnosis and to verify the possible relationship between host, slum environment and RHD. | Cross section | 4,848 students aged 5-16 years  Kinshasa, Democratic Republic of Congo | Persons in household >8 | RHD diagnosed on echocardiography of suspect cases | 14.03/ 1,000 |  | **Positive association**  >8 persons in household OR 4.10 (1.70-9.85) | Fair: limited results presented. |
| McLaren et al 1975 | An epidemiological survey to define the exact magnitude of the problem of RHD in black children in the crèches and schools of Soweto. | Cross section | 12,050 black children aged 2-18 years  Soweto, South Africa | Number of siblings >3 | RHD diagnosed by clinical examination.  Throat swab culture for GAS | 6.9 /1,000 | **Positive association** Number of siblings >3 (χ^2^ =5.18, p<0.05) |  | Poor: outcome ascertainment method poor, no multivariate analysis. |
| Piper 1970 | To investigate whether the increased incidence of ARF is associated with residence in a municipality of W. rather than with Mennonite ethnicity. | Cross section | 193 families with school age child in area of high ARF incidence (municipality of W) and low ARF incidence  Saskatoon, Canada | Floor area of all living rooms in the house < 400 feet^2^  ≥2 persons per bedroom | ARF diagnosed using Duckett-Jones criteria | ARF in municipality W. 2.3/1000 | **Possible association** Residents of W. vs. other areas:  < 400 ft^2^ living space 83 (5.7 > expected) vs. 80 (5.7 < expected)  ≥2 per bedroom 57 (11.5 > expected) vs. 33 (11.5 < expected)  (no test of significance) |  | Poor: limited analysis, crude measure of space. |
| Poppi et al 1953 | To ascertain the prevalence of ARF and RHD in a region of Italy where the high incidence of both complaints is well known to practicing physicians, and to evaluate the weight of some factors generally admitted as important in the pathogenesis of the disease. | Cross section | 930 female manual labourers aged 14-70 years  Po valley, Italy | Crowded living accommodation (not defined further) | ARF based on clinical history of acute migrating polyarthritis, confining patient to bed with fever and subsiding after salicylates, or of Sydenham’s chorea.  RHD diagnosed with examination, orthodiagram of the heart and ECG. | 14.9% history of ARF or chorea  8.7% RHD | **No association**  Crowded living |  | Poor: ill-defined exposure and poor presentation of results. |
| Quinn et al 1948 | To select and examine comparable rural and urban populations from a single small area of the US and to determine the rates for RHD in each population, and to answer: what is the effect of living conditions within high and low rental areas in cities; what affect does crowding in the home have on the prevalence of RHD; and what is the familial incidence of ARF and RHD in this geographic area. | Cross section | 3,141 children rural, semi-urban and urban based, aged 11 to 15 years  Connecticut, USA | Crowding=  Urban home: <1 room per person  Rural homes: number of rooms 2 < number of occupants | ARF history based on clinical history of rheumatic fever or chorea.  RHD diagnosed using criteria based on clinical examination | 2.1% history ARF  2.1% RHD | **Positive association**  Crowding 34.4% ARF or RHD vs. 24.1% non-ARF, non-RHD (χ^2^=6.25, p=0.044) |  | Poor to fair: Poor case ascertainment method, no multivariate analysis. Well described study. |
| Quinn et al 1950 | To check the validity of the high RHD rate in Ansonia and to analyse factors which might influence the prevalence of RHD within that community. | Cross section | 1,229 children aged 10 to 18 years  3 industrial cities and one non-industrial city in Connecticut, USA | >1 persons per room  >2 persons per bedroom  >2 persons per bed | RHD diagnosed on clinical examination findings | 4.6% | **Positive association**  In non-industrial area:  >1 persons per room  58.3% RHD vs. 17.68% non-RHD (χ^2^=12.7, p<0.0001)  **No association**  In industrial area:  >1 per room  >2 per bedroom  >2 per bed  In non-industrial area:  >2 per bedroom  >2 per bed |  | Poor: Poor case ascertainment method, no multivariate analysis. |
| Rizvi et al 2004 | To determine the prevalence of RHD in a rural population in a single subdistrict and study the risk factors for RHD. | Cross section | 10,412 participants interviewed & 9,483 screened across  11 rural villages  Pakistan | Crowding index:1-3  1= single room with ≥5 people  3= 3 rooms with <5 people or > 3 rooms) | ARF diagnosed using updated 1992 Jones criteria  RHD diagnosed using echocardiography for cases with clinical murmurs | RHD 5.7/ 1000 | **No association** Crowding index |  | Fair to good: generally good methods, unconventional measure for crowding index. |
| Saxena et al 2011 | To estimate the prevalence of clinical and subclinical RHD, to identify risk factors associated with RHD and to study the natural history of children with echocardiographically detected RHD. | Cross section | 6,270 school children aged 5-15 years  Rural area,  North India | Crowded household >4 persons per room | RHD diagnosed using modified WHO criteria of echocardiogram. | 20.4/1000 | **Positive association**  RHD cases per 1000: 28.3 crowded households vs. 18.6 uncrowded households (p<0.05) | **No association**  Crowed household | Fair: no power calculation. |
| Spitzer et al 2001 | To compare the carriage rate of group A streptococcus in the Orthodox Jewish population in north Hackney. | Cross section | 1,223 participants aged over 2  UK | Crowding index= number of children+1/number of bedrooms | Throat swab for GAS | 5% GAS carriage rate in Orthodox Jews | **Positive association**  Crowding index OR 1.95 (1.26-3.01) | **No association**  Crowding index | Good: adequately powered and undertook multivariate analysis. |
| Vashistha et al 1993 | To find out the magnitude of the problem of RHD in children and to make an early diagnosis to prevent further complications. | Cross section | 8,449 school children aged 5-15 years  Agra, India | Family size <5 or >10 | ARF diagnosed using revised Jones criteria.  RHD diagnosed on clinical, radiographic, ECG and echocardiographic findings | RHD 1.4/1000 | **Possible association**  RHD prevalence in family size <5  0.11% vs. family size >10 0.28%  (no test of significance) |  | Poor: poor reporting of results and statistical analysis. |
| Yazov et al 1978 | To present the results of the first stage of a prospective epidemiological study of GAS and RHD. | Cross section | 1,012 school children aged 6-20 years  Addis-Ababa, Ethiopia | Number of persons living in one room | RHD diagnosed on clinical findings.  Throat swab culture | 4.24% swabs GAS +  RHD 4.9 /1000 | **No association**  Mean persons per room |  | Poor: poor description of methods and analysis. |
| Gordis et al 1969 | To explore the relationship of socio-economic status to incidence rates of rheumatic fever to determine whether socio-economic differences can adequately account for ethnic differences in incidence. | Ecologic | Number not reported.  Aged 5-19 years  Baltimore, USA | Crowding= % of housing units with >1 persons per room  Ecologic unit: 168 census tracts | ARF diagnosed using hospital medical records between 1960 and 1964 | Various. From 3.4 per 100,000 among whites in highest socioeconomic fifth to 26.6 per 100,000 in blacks in lowest socioeconomic fifth. | **Possible association** Crowding index quintile and ARF incidence per 100,000 in white population:   - 0.6-2.4%: 4.6 - 2.5-3.6%: 4.2 - 3.7-5.2% 7.5 - 5.3-8.2%: 9.8 - 8.3-25.3%: 29.9   In non-white population:   - 0-16.3%: 19.1 - 16.4-20.9%: 22.3 - 21.3-23.8%: 24.5 - 23.9-27.5%: 29.0 - 27.7-50.9%: 19.7 |  | Fair: limited confounders included, no multivariate analysis. |
| Jaine et al 2011 | To test the hypothesis that household crowding was positively associated with ARF incidence and whether there was a dose-response relationship between the exposure and ARF risk. | Ecologic | 1,249 ARF cases between 1996 and 2005.  New Zealand | Crowding measured using Canadian occupancy standard | ARF diagnosed from hospital recorded diagnosis | Average annual rate ARF 3.4/ 100,000 | **Positive association**  Rate ratio of ARF cases /100,000 by crowding quintile   1. 1.0 (reference) 2. 1.8 (1.1-2.9) 3. 2.9 (1.8-4.6) 4. 5.7 (3.6-8.9) 5. 23.1 (15.0-35.6) | **Positive association**  Proportion of crowded households: IRR 1.065 (1.052-1.079) | Good: well described study, consistent and valid exposure and outcome measures. |
| McDonald et al 2007 | To investigate the epidemiology of symptomatic pharyngitis and pyoderma in a Central Australian community with known high rates of RHD and compare to Top End communities using essentially the same surveillance methods. | Ecologic | 91 children and 45 adults from 13 households with history of either an ARF or RHD case  Remote community, Australia | Median number of people per bedroom in community based on government survey of household numbers from 2004 | Throat and wound swab culture and typing |  | **Possible association**  Throat carriage rates 3.7% Central Australian community vs. 4.5% Top End Community  Crowding: 1.7 Central Australian community vs. 6.9 Top End Community  (no test of significance) |  | Poor: attrition not reported, no direct test of association. |
| Morton & Lichty 1970 | To describe the evidence which suggests the existence of a region within Colorado in which excess risks of occurrence of rheumatic fever were associated with socioeconomic factors manifest in 1959-61. | Ecologic | 75 cases RHD  Colorado, USA | Persons per household | ARF cases and ARF/RHD death data from Colorado Department of Public Health | Varied by region. Average mean annual rate 14.0/100,000; range 7.6 to 64.6 /100,000 | **Possible association** Average persons per household in region of highest ARF rates vs. state average 3.7 vs. 3.21  (no test of significance) |  | Poor: poor analysis, no multivariate. |
| Perry & Roberts 1937 | To further study the very high incidence of ARF and RHD in Bristol compared to surrounding counties. | Ecologic | 754 RHD cases  Bristol UK | Persons per room based on census data 1921 and 1931. | RHD reported by private practitioners and school medical officers | 0.21/1,000 | **Positive association**  RHD cases increased by 55 per 100,000 for every increase of 0.1 in density of persons per room.  Density of persons per room accounted for >40% of variation in incidence of RHD (p<0.05) |  | Poor to fair: no multivariate analysis, crude outcome ascertainment |
| Phillips & Osmond 2014 | To investigate the possible role of developmental influences in RHD | Ecologic | 37,321 deaths from RHD between 1968-78  UK | Data from 1921 census by borough:  Number of rooms  Number of families  Family size  Number of rooms per person  Number of people per acre | RHD death identified on death certificates | Deaths from RHD 33.86/100,000. | **Positive association**  Correlation to RHD death:  Population size (r=0.17, p<0.05)  Persons per acre (r=0.51, p<0.001)  Rooms per house (r=-0.43, p<0.001)  Families per house (r=0.24, p<0.001)  Persons per family (r=0.40, p<0.001)  Rooms per person (r=-0.69, p<0.001) | **Positive association**  Model 1- with infant diarrhoea mortality  Persons per room RR 1.072 (1.052-1.092)  Persons per family RR 1.032 (1.018-1.045)  Model 2- with infant bronchiolitis mortality  Persons per room RR 1.071 (1.050-1.092)  Persons per family RR 1.019 (1.005-1.032) | Fair: may be incomplete outcome ascertainment, limited confounders explored. |
| Roberts et al 2015 | To describe the prevalence of definite and borderline RHD in Indigenous children in different regions of Australia and the Torres Strait and to inform decision making about the potential impact and usefulness of echocardiographic screening for RHD in different Australian regions. | Ecologic | 3,964 Indigenous children aged 5-14 years in four different regions  Australia | Mean number of people per household in each community based on national 2011 census. | RHD diagnosed using World Heart Foundation echocardiographic criteria for all participants. | Various. From 4.7 per 1000 in Far North Queensland to 15 per 1000 in Top End region. | **Possible association**  Number of people per household higher in the Top End community compared to others (6.3 vs. 4.8, 4.5, 4.8), which followed RHD prevalence (15.0/1000 vs. 6.7, 4.7, 8.9/1000)  (no test of significance) |  | Poor to fair: low participation rate, no test of significance of SES factors and RHD prevalence. |
| Wedum et al 1944 | To examine all hospital admissions of cases of rheumatic fever in Cincinnati between 1930-1940. | Ecologic | 517 ARF cases of all ages with or without RHD  Cincinnati, USA | Crowding: >11% houses in census tract with >1.5 persons per room  High density: >22 persons per acre | ARF diagnosed from hospital admission records  High prevalence census tract= census tracts with ≥10 cases per 100,000  Low prevalence census tract= census tracts with <10 cases per 100,000 |  | **Positive association**  Crowding: 25 high prevalence tracts vs. 1 low prevalence tracts(X^2^=61.8)  High density: 30 high prevalence tracts vs. 19 low prevalence tracts (X^2^=45.0) | **Positive association**  Crowding + high density 24 high prevalence tracts vs. 2 low prevalence tracts (X^2^=54.5)  (Adjusted for mean monthly rental <$21.65 & >5% negroes) | Fair: criteria for diagnosis not reported, statistical methodology not robust |

ARF: Acute rheumatic fever ASOT: Anti-streptolysin O titre βHS: Beta haemolytic streptococci ECG: Electrocardiogram emmST: emm sequence type HR: Hazard ratio ICD: International Classification of Diseases IRR: Incidence rate ratio GaβHS: Group A beta haemolytic streptococci GAS Group A streptococci NA: Not applicable OR: odds ratio RHD: Rheumatic heart disease RR: Risk ratio UK: United Kingdom USA: United States of America WHO: World Health Organization
