# Supplementary material for: The role of social determinants of health in the risk and prevention of group A streptococcal infection, acute rheumatic fever and rheumatic heart disease: A systematic review
Source: PLoS Negl Trop Dis. 2018 Jun 13;12(6):e0006577. doi: 10.1371/journal.pntd.0006577 (PMC6016946; doi:10.1371/journal.pntd.0006577)
Supplement: S3 Table — (DOCX) [file pntd.0006577.s006.docx]

S3 Table. Summary of dwelling characteristics and facilities and GAS infection, ARF and RHD

| Study details | Aim of study | Study design | Study population and setting | Measure of dwelling type or dwelling characteristics | Measure of outcome (GAS, ARF, RHD) | Outcome incidence/ prevalence | Results univariate | Results: multivariate | Study quality |
| --- | --- | --- | --- | --- | --- | --- | --- | --- | --- |
| Adanja et al 1988 | To test the hypothesis regarding the influence of socioeconomic and some other factors on occurrence of ARF. | Case control | 148 with first ARF attack, 444 controls matched for age, sex and place of residence (1:3)  Serbia | Condition of the dwelling: good or deteriorated  Location: ground floor or basement  Household facilities: courtyard/terrace, toilet, water  Household characteristics: sunny home, damp home. | ARF using revised Jones criteria | NA | **Positive association**  Deteriorated home 25.7% vs. 17.1%, RR 1.83 (p=0.0100)  Damp home 15.5% vs. 7.2%, RR 2.48 (p=0.0042)  **No association**  Location, courtyard/terrace, toilet, water, sunny home. |  | Poor to fair: cannot determine temporal association of exposure and outcome; no multivariate analysis. |
| Dobson et al 2012 | To investigate the role of environmental factors for RHD in Fiji. | Case control | 80 children aged 5–15 years with RHD and 80 age and sex matched controls  Fiji | Dwelling type: nonconcrete or concrete  Electricity or no electricity  Cooking method: wood or electric/gas  Ventilation for cooking or no ventilation | Definite RHD diagnosed on echocardiogram using WHO criteria | NA | **No association**  Dwelling type, electricity, cooking method, ventilation |  | Poor: no power calculations, unstated number of controls from different source, participation rate 61%. |
| Grave 1957 | To investigate the factors of social and emotional forces in the aetiology of rheumatic fever. | Case control | 122 children aged 2–12 with ARF and 100 controls from outpatient clinics within same age range  Sydney, Australia | Substandard housing: tenement, slum or tent.  Damp house: obvious mildew, permanently leaking roofs, high-water table.  Sewerage/drainage | ARF diagnosed on criteria of the Rheumatic fever council of the American Heart Association | NA | **Positive association**  Substandard housing 15% vs. 4%, OR 4.2 (1.32-17.71)  Damp house 26% vs. 13%, OR 2.33 (1.09-5.17) *  **No association**  Sewerage/drainage |  | Poor: unmatched controls and no adjusting for differences, no power calculations, no test of significance |
| Gray et al 1952 | To follow up a previous study with a long period of observation to compare rheumatic and control families with respect to hereditary and certain environmental factors which might be responsible for the familial prevalence of rheumatic fever. | Case control | 40 families with a ARF case and 30 control families with a scarlet fever case  Connecticut, USA | Housing criteria from Office of Rent Control: Substandard (no central heating, shared toilets, in need of major repairs), Passable (central heating, private toilets), Good (good construct and condition) | ARF diagnosed by physicians at time of acute episode (criteria not reported).  RHD diagnosed using clinical criteria of the New York and American Heart Association | NA | **Positive association**  Substandard housing ‘highly significance difference’ 93.4 vs. 70.6%  (statistical test not reported) |  | Poor: high proportion of cases lost to follow up, test of significance not reported |
| Hewitt & Stewart 1952 | This study deals with the social background of acute rheumatism. | Case control | 793 children aged 5-14 years  Sheffield, UK | Condemned house  Degree of cleanliness: good, fair, poor.  Type of house: detached, semi-detached , terrace, pre-fab, court, back-to-back, other.  Dampness: none, floor, walls and ceiling.  Light: good, fair, poor  Ventilation: good, fair, poor  Structure: good, fair, poor | Acute rheumatism diagnosis based on notification criteria of the County Borough | NA | **No association**  Condemned  Degree of cleanliness  Type of house  Dampness  Light  Ventilation  Structure |  | Poor: no baseline comparison, numbers not reported, participation rate not reported. |
| Riaz et al 2013 | To identify the risk factors of ARF and to explore the risk factors for developing RHD among ARF patients | Case control | 103 RHD cases, 103 ARF and 207 controls  Bangladesh | Wall material: Semi-*pucca*/*kaccha*, *Pucca*/Brick  Water supply: supply/surface water, tubewell/groundwater  Bed: *Khat*, floor | ARF diagnosed using modified Jones criteria  RHD diagnosed by doppler echocardiography | NA | **Positive association**  For ARF:  *Pucca*/Brick OR 2.3 (1.4-3.7)  Tubewell/groundwater Control OR 0.3 (0.29-0.6)  For RHD:  *Pucca*/Brick OR 1.8 (1.2-3.0)  Tubewell/groundwater  RHD OR 0.55 (0.34-0.9)  **No association**  For ARF & RHD  Bed | **Positive association**  For ARF:  *Pucca*/Brick OR 3.6 (1.6-8.1)  For RHD:  *Pucca*/Brick OR 2.8 (1.3-5.3)  **No association**  For ARF & RHD  Tubewell/groundwater  Bed  For RHD in ARF cases  *Pucca*/Brick  Tubewell/groundwater  Bed | Fair: no matching, blinding of assessors not stated. |
| Vlajinac et al 1991 | To investigate the independent, unconfounded effect of risk factors for ARF identified in a previous study conducted on this population. | Case control | 148 with first ARF attack and 444 controls matched for age, sex and place of residence (1:3)  Serbia | Home dampness | ARF diagnosed using revised Jones criteria | NA |  | **Positive association**  Home dampness RR 2.40 (1.26-4.58) | Fair: temporal association of exposure and outcome not explicitly stated; |
| Vlajinac et al 1989 | To test the hypothesis that socio-economic factors are related to rheumatic fever and make an additional comparison for those with and without a history of sore throat. | Case control | 148 with first ARF attack and 444 controls matched for age, sex and place of residence (1:3) | Home dampness | ARF diagnosed using revised Jones criteria  Frequent sore throat= >1/year | NA | **Positive association**  For participants with sore throat:  Home dampness RR 3.56 (1.79-7.07)  **No association**  For participants without sore throats: Home dampness |  | Poor to fair: temporal association of exposure and outcome not explicitly stated; stratified only by sore throat frequency. |
| Zaman et al 1997 | To examine data on patients with proven GAS infection presenting to a ARF hospital to identify socio-economic factors that may need further exploration. | Case control | 44 ARF cases and 86 controls aged 5-20 years with recent GAS infection  Dhaka, Bangladesh | Housing construction: *Paka* (bricks, concrete) or *kacha* (other materials) | ARF diagnosed on revised Jones criteria | NA | **Positive association**  *Kacha* 34% vs. 15% (p=0.01) | **Position association**  *Kacha* OR 2.93 (1.23-7.15) | Fair to good: no power calculations |
| Zaman et al 1998 | To explore further the nutritional factors that may be associated with ARF. | Case control | 60 ARF cases and 104 controls aged 5-20 years with recent GAS infection  Dhaka, Bangladesh | Proportion without brick house | ARF diagnosed using updated Jones criteria | NA | **Positive association**  Proportion without brick house 53.5% vs. 20.2% (p<0.0001) |  | Fair to good: no power calculations. |
| Bach et al 1996 | To report how the Martinique/Guadeloupe ARF eradication programme was set up and its results over 10 years. | Case series | Not stated  French Caribbean | Basic household facilities: mains water, toilets and electricity. | ARF diagnosed using modified Jones criteria | 19.6/ 100,000 residents <20 years | **Possible association**  Basic household facilities 42% ARF families vs. 58% general population (no test of significance) |  | Poor: limited analysis and incomplete reporting of figures |
| Coggon et al 1993 | To examine the associations with overall mortality and specific diseases (RHD) that might be expected to relate to specific aspects of housing. | Cohort | 51 deaths from RHD between 1951-1989 | Household facilities: hot water tap, purpose built larder, Gas cooker | Cause of death listed as RHD on Death Certificate |  | **No association**  Household facilities |  | Poor: unreliable measures of exposures and outcome, sampling method not reported. |
| Mirabel et al 2015 | To address the outcomes and modalities of RHD screening through a cohort of children with and without RHD who took part in the first large RHD echocardiography based surveillance programme. | Cohort | 114 cases of RHD from cohort of 157 and 227 controls selected randomly from classmates, matched for ethnicity and classroom  New Caledonia | Housing construction: Concrete, part-concrete, non-concrete | Persistence of RHD diagnosed using World Heart Foundation criteria.    ARF diagnosed using Australian Guideline for Prevention, Diagnosis and Management of ARF and RHD. | RHD: 890/100,000  ARF: 10.28/1000/year | **No association**  Housing construction |  | Fair: no power calculation, >20% lost to follow up. |
| Nandi et al 2001 | To estimate the incidence and risk factors for GAS sore throat among school-aged children in northern India | Cohort | 536 children aged 5-15 in 26 peri-urban slum households  Chandigarh, India | Separate kitchen area in house | Throat swab culture for GAS | 1 episode/child/year among 5-15 year olds. | **Positive association**  Incidence of GAS per child year in no kitchen 0.99 vs. kitchen 0.87 (p<0.01) |  | Poor to fair: no power calculations, no multivariate analysis. |
| Tay et al 1981 | To collect information pertaining to the following points: the carriership of beta-haemolytic streptococcal throat and skin diseases; the incidence of various serological groups of haemolytic streptococci and of the types of GAS; the role played by the various factors like race, season of the year, socio-economic status etc. on the clinical, epidemiological and microbiological patterns of the streptococcal carriership and of the disease; the assessment of the best therapy regime for streptococcal disease applicable in the local conditions. | Cohort | 491 primary school students  Singapore | Type of housing:  Attap/Zinc, Housing Development Board flat, Private housing | Throat and skin swab cultures | Overall cumulative incidence of GAS throat carrier rate was 46.7% | **Positive association**  GAS cumulative incidence in Attap/zinc 74.5%, Housing Development Board flats 44.8%, private housing 18.9%. χ^2^=83.44 (p<0.0001)  GAS cumulative incidence in Attap/zinc and Housing  Development Board vs. Private Housing RR 2.98 (2.05-4.32)* |  | Poor to fair: >20% attrition, no power calculation, poor reporting of statistics. |
| Ba-Saddik et al 2011 | To estimate the prevalence of RHD among school-children aged 5-16 years in Aden (Yemen). | Cross section | 6,000 school children aged 5-16 years  Aden, Yemen | Housing condition:  Good ( made of stone, >4 rooms, water supply) Satisfactory (made of stone, water supply, but small)  Bad (constructed of other materials, no water supply) | Definitive RHD diagnosed using modified Duckett Jones’ criteria and WHO criteria for Doppler abnormalities, in those with clinical murmurs. | 36.5/1000 children | **Positive association**  Bad housing 39.3% RHD vs. 21.6% non-RHD children (χ^2^ for trend (p=0.001) |  | Poor to fair: no adjustment, non-blinded assessors |
| Likitnukal et al 1994 | To evaluate the factors influencing streptococci colonization of school age children. | Cross section | 1,547 school children aged 6-11 years  Bangkok, Thailand. | Stagnant water underneath the house | Throat swab culture for βHS and GAS | GAS 18%  βHS 47% | **No association**  Stagnant water underneath |  | Poor: high attrition, no power calculation. |
| Poppi et al 1953 | To ascertain the prevalence of ARF and RHD in a region of Italy where the high incidence of both complaints is well known to practicing physicians, and to evaluate the weight of some factors generally admitted as important in the pathogenesis of the disease. | Cross section | 930 female manual labourers aged 14-70 years  Po valley, Italy | Damp house | ARF based on clinical history of acute migrating polyarthritis, confining patient to bed with fever and subsiding after salicylates, or of Sydenham’s chorea.  RHD diagnosed with examination, orthodiagram of the heart and ECG. | 14.9% history of ARF or chorea  8.7% RHD | **Positive association**  Damp house significantly correlated with history of rheumatic fever χ^2^=26.4 (p<0.005) |  | Poor: ill-defined exposure measures and poor presentation of results. |
| Quinn et al 1950 | To check the validity of the high RHD rate in Ansonia and to analyse factors which might influence the prevalence of RHD within that community. | Cross section | 1,229 children aged 10-18 years  3 industrial cities and one non-industrial city in Connecticut, USA | Dwelling standard:  Inadequate (no central heating with shared toilet facilities and need for major improvements)  Passable (most homes with central heating, private toilets and need to major repairs met)  Adequate to good (good construction and repair) | RHD diagnosed on clinical examination findings | 4.6% RHD | **No association**  Dwelling standard |  | Poor: Poor case ascertainment method, no multivariate analysis. |
| Rizvi et al 2004 | To determine the prevalence of RHD in a rural population in a single subdistrict and study the risk factors for RHD. | Cross section | 10,412 participants interviewed & 9,483 screened across  11 rural villages  Pakistan | Housing construction  *Pucca*, Semi *pucca* or *kutcha* | ARF diagnosed using updated 1992 Jones criteria  RHD diagnosed using echocardiography for cases with clinical murmurs | RHD 5.7/ 1000 | **No association**  Housing construction |  | Fair to good: generally good methods, unconventional measure for crowding index. |
| Rushdy et al 1995 | To investigate an outbreak of GAS in a boarding school of 261 pupils and 45 staff. | Cross section | 133 screened  Boarding school, UK | Dormitory type: poorly ventilated | Nose and throat swabs for GAS | GAS attack rate in boarding school 16% | **Positive association**  Attack rate in poorly ventilated dormitories 29% vs. well ventilated dormitories 12%, OR 3.47, χ^2^=5.74 (p=0.02) |  | Poor: small numbers, no confounders explored. |
| Saxena et al 2011 | To estimate the prevalence of clinical and subclinical RHD, to identify risk factors associated with RHD and to study the natural history of children with echocardiographically detected RHD. | Cross section | 6,270 school children aged 5-15 years  Rural area,  North India | Housing construction: *Kutcha* | RHD diagnosed using modified WHO criteria of echocardiogram. | 20.4/ 1000 | **Positive association**  Living in *kutcha* house (p<0.005) (figures not reported) | **No association**  *Kutcha* house | Fair: no power calculation. |
| Rosati et al. 1978 | To investigate the frequency of MS, rheumatoid arthritis, RHD and post-streptococcal nephritis in a population ethnically homogeneous and stable in size and composition, exclusively on the basis of differences in climatic and socioeconomic conditions. | Ecologic | 813 cases of RHD | Houses without toilet and drinkable water | RHD diagnosed on hospital records of heart disease as a clinical manifestation of rheumatic fever diagnosed using revised Jones criteria | Various. Range 0.42 to 0.89/ 1000 | **Possible association**  Regions with highest proportion without toilet and drinkable water had highest frequency of RHD  Zone 1: 20%, 0.89/1000  Zone 2: 10%, 0.76/1000  Zone 3: 1.7%, 0.42/1000  Zone 4: 0.5%, 0.46/1000  (no test of significance) |  | Poor: ecologic unit too broad, no direct analysis. |
| Falck et al 1998 | To test the hypothesis that treatment failures of streptococcal pharyngotonsillitis may be caused by reinfection by the patients’ own streptococci remaining on a toothbrush or in the bedclothes. | Randomised control trial | 50 patients in intervention group, 64 in control group | Intervention: written hygiene instructions to change bed clothes, toothbrush, ash toys and dummies.  Control: No instructions given.  Sweden | Nose and throat swabs for GAS day 0, 6–10, 28–35.  Environmental swabs | GAS recurrence after 35 days 11.4% | **No association**  Hygiene instructions and recurrence |  | Poor: errors in testing methods and treatment protocol, non-blinded, poor adherence to study. |
| Luby et al 2005 | To measure the broad health benefits brought about by improvement of handwashing and bathing with soap in settings where communicable diseases are leading causes of childhood morbidity and mortality. | Randomised control trial | 1,523 children under 15 years intervention 1, 1,640 children intervention 2 and 1,528 children in control group  Squatter settlements, Karachi  Pakistan | Intervention 1: Hand washing promotion and antibacterial soap  Intervention 2: Hand washing promotion and plain soap  Control: no specific hand washing promotion. | Impetigo diagnosed as new skin eruption confirmed by study physician | Range mean impetigo incidence: 0.61–0.94 episodes/100 person-weeks | **Positive association**  Difference in mean impetigo incidence vs. control neighbourhoods:  Antibacterial soap -36% (-53% – -18%)  Plain soap -34% (-52%– -16%) |  | Good: well designed and powered study, double blind for intervention arm. |
| Perry 1957 | To test the infectivity of streptococci deposited on blankets, naturally contaminated blankets and freshly laundered blankets were issued through regular military channels to men living in 1 of 4 barracks during the winter of 1952. | Randomised control trial | 85 army recruits in intervention group, 112 in control group.  Air Force Base,  US | Intervention: issued blankets obtained from men harbouring GAS in their nose | Nose and throat swabs for GAS at day 0, then 1-2 times weekly. 2 consecutive cultures were positive.  Blankets swabbed for GAS |  | **No association**  GAS acquisition or GAS infection and GAS contamination of blanket |  | Fair: single blinded, no baseline characteristics reported, no power calculation |

*Test of significance calculated for systematic review from original study data

ARF: Acute rheumatic fever βHS: Beta haemolytic streptococci ECG: Electrocardiogram emmST: emm sequence type GAS Group A streptococci NA: Not applicable OR: odds ratio RHD: Rheumatic heart disease RR: Risk ratio UK: United Kingdom USA: United States of America WHO: World Health Organization
