# Supplementary material for: The role of social determinants of health in the risk and prevention of group A streptococcal infection, acute rheumatic fever and rheumatic heart disease: A systematic review
Source: PLoS Negl Trop Dis. 2018 Jun 13;12(6):e0006577. doi: 10.1371/journal.pntd.0006577 (PMC6016946; doi:10.1371/journal.pntd.0006577)
Supplement: S4 Table — (DOCX) [file pntd.0006577.s007.docx]

S4 Table. Summary of education and GAS infection, ARF and RHD

| Study details | Aim of study | Study design | Study population and setting | Measure of parental education | Measure of outcome (GAS, ARF, RHD) | Outcome incidence/ prevalence | Results univariate | Results Multivariate | Study quality |
| --- | --- | --- | --- | --- | --- | --- | --- | --- | --- |
| Adanja et al 1988 | To test the hypothesis regarding the influence of socioeconomic and some other factors on the occurrence of ARF. | Case control | 148 with first ARF attack, 444 controls matched for age, sex and place of residence (1:3)  Serbia | Low education of mother: < 4 years elementary school education  Low education of father: < 4 years elementary school education | ARF using revised Jones criteria | NA | **Positive association**  Low education of mother 18.9% vs. 10.4% RR 2.58 (1.38-4.83)  **No association**  Low education of father |  | Poor to fair: cannot determine temporal association of exposure and outcome; no multivariate analysis. |
| Dobson et al 2012 | To investigate the role of environmental factors for RHD in Fiji. | Case control | 80 children aged 5- 15 years with RHD and 80 age and sex matched controls  Fiji | Maternal education: Primary school, Secondary school | Definite RHD diagnosed on echocardiogram using WHO criteria | NA | **No association** Maternal education level |  | Poor: no power calculations, unstated number of controls from different source, participation rate 61%. |
| Grave 1957 | To investigate the factors of social and emotional forces in the aetiology of rheumatic fever. | Case control | 122 children aged 2-12 with ARF, 100 controls from outpatient clinics within same age range  Sydney, Australia | Years of maternal education | ARF diagnosed on criteria of the Rheumatic fever council of the American Heart Association | NA | **No association**  Years of maternal education |  | Poor: unmatched controls and no adjusting for differences, no power calculations, no test of significance |
| Riaz et al 2013 | To identify the risk factors of ARF and to explore the risk factors of developing RHD among ARF patients. | Case control | 103 RHD cases, 103 ARF cases, 207 controls  Bangladesh | Education level of mother and father: Secondary and above, Primary or less, Illiterate | ARF diagnosed using modified Jones criteria  RHD diagnosed by doppler echocardiography | NA | **Positive association** Mother illiterate: non-ARF 33.8% (reference), ARF 46.6% (χ^2^6.7 p=0.033), RHD 50.5% (χ^2^11.7 p=0.003)  **No association**  Education level of father |  | Fair: no matching, blinding of assessors not stated. |
| Kurahara et al 2006 | To determine factors in prevalence rates of ARF in a multiethnic population. | Case control | 26 cases ARF, 41 controls with other heart condition (all on Medicaid)  Hawaii, USA | Mother or father with a high school diploma | ARF diagnosed using modified Jones criteria | NA | **No association** Mother or father with a high school diploma |  | Fair: no matching, small sample size |
| Okello et al 2012 | To investigate the role of socioeconomic and environmental factors in the pathogenesis of RHD in Ugandan patients. | Case control | 243 RHD cases, 243 controls aged between 5 and 60  Uganda | Education level of case: no education, Primary, Secondary, Vocational, University | RHD diagnosed using history ARF, clinical examination, echocardiogram criteria | NA | **Positive association** Education level Primary or less 53.9% vs 25.9% OR 3.34 (2.24-4.99) | **No association**  Education level | Fair: unmatched controls, but randomly chosen and multivariate analysis performed, time period of participant selection not stated. |
| Vlajinac et al 1991 | To investigate the independent, unconfounded effect of risk factors for ARF identified in a previous study conducted on this population. | Case control | 148 with first ARF attack, 444 controls matched for age, sex and place of residence (1:3)  Serbia | Low education of mother: < 4 years elementary school education | ARF diagnosed using revised Jones criteria | NA |  | **Positive association**  Low education of mother RR 2.52 (1.29-4.92) | Fair: temporal association of exposure and outcome not explicitly stated; |
| Vlajinac et al 1989 | To test the hypothesis that socio-economic factors are related to rheumatic fever and an additional comparison for those with and without a history of sore throat. | Case control | 148 with first ARF attack, 444 controls matched for age, sex and place of residence (1:3)  Serbia | Low education of mother < 4 years elementary school education | ARF diagnosed using revised Jones criteria  Frequent sore throat= >1/year | NA | **Positive association**  Without sore throat: Low education of mother RR 2.23 (1.16-4.25)  **No association**  With sore throat: Low education of mother |  | Poor to fair: temporal association of exposure and outcome not explicitly stated; stratified only by sore throat frequency. |
| Zaman et al 1998 | To explore further the nutritional factors that may be associated with ARF. | Case control | 60 ARF cases, 104 controls aged 5-20 years with recent GAS infection  Dhaka, Bangladesh | Parental schooling in years | ARF diagnosed using updated Jones criteria | NA | **Positive association** Parental schooling 10.3 vs. 17.8 (p<0.0001) |  | Fair to good: no power calculations. |
| Meira et al 2005 | To study the progress of valvar disease by means of clinical and echocardiographic evaluations and to identify the independent variables that predict severe chronic valvar disease. | Case series | 258 children and adolescents diagnosed with ARF between 1983 to 1998  Belo Horizonte, Brazil | Mother’s schooling: <4 years | ARF diagnosed using revised Jones criteria |  | **No association** Mother’s schooling | **Positive association** Mother’s schooling < 4 years RR 3.93 (1.46-10.57) | Fair: reasonable breadth of factors, multivariate analysis, no power calculation |
| Mirabel et al 2015 | To address the outcomes and modalities of RHD screening through a cohort of children with and without RHD who took part in the first large RHD echocardiography based surveillance programme. | Cohort | 114 cases of RHD selected from a cohort of 157 and 227 controls selected randomly from classmates, matched for ethnicity and classroom  New Caledonia | Mother’s education: Primary, Secondary,  Higher education | Persistence of RHD diagnosed using World Heart Foundation criteria. | RHD: 890/100,000 | **Positive association** Mother’s education:  Primary 46.3% vs 54.2%  Secondary 50% vs. 29.2%  Higher 3.7% vs. 16.7% (p=0.048) | **No association** Mother’s education | Fair: no power calculation, >20% lost to follow up. |
| Bassili et al 2000 | To evaluate the current regimen of secondary prophylaxis available to disadvantaged Egyptian children suffering from RHD who were attending various children’s hospitals in Alexandria. | Cross section | 150 children with RHD | Maternal education: ≤6 years, >7 years  Paternal education: ≤6 years, >7 years | Diagnosis of RHD based on a documented previous history of ARF with characteristic murmur confirmed by Doppler echocardiography | NA | **No association**  Maternal or paternal education |  | Poor to fair: no power calculation, limited factors included |
| Likitnukal et al 1994 | To evaluate the factors influencing streptococci colonization of school age children. | Cross section | 1,547 school children aged 6-11 years  Bangkok, Thailand | Maternal and paternal education levels:  ≤Lower primary school  ≤ Upper primary school  ≤ Secondary school | Throat swab culture for βHS and GAS | GAS 18%  βHS 47% | **No association** Maternal or paternal education levels |  | Poor: high attrition, no power calculation. |
| Rizvi et al 2004 | To determine the prevalence of RHD in a rural population in a single subdistrict and study the risk factors for RHD. | Cross section | 10,412 participants interviewed & 9,483 screened across  11 rural villages  Pakistan | Education among patients:  Illiterate  Able to read or having any formal education | ARF diagnosed using updated 1992 Jones criteria  RHD diagnosed using echocardiography for cases with clinical murmurs | RHD  5.7/ 1000 | **No association**  Patient education level |  | Fair to good: generally good methods, unconventional measure for crowding index. |
| Lue et al 1979 | To collect information on streptococcal infections, prevalence and severity of ARF and RHD and their long term follow up. | Ecologic | Various | Literacy rates among urban and “well to do children” | GAS diagnosed on throat swab culture  Diagnosis of ARF was made based on the revised or modified Jones criteria.  RHD diagnosis from hospital record | Various. Range for GAS 1.6-29.5%  Range for RHD 0.3 to 2.7/ 1,000 | **No association**  Literacy rates and GAS  Literacy rates and RHD |  | Poor: heterogeneity in methods, populations and results. Ill-defined ecologic units. |
| Morton & Lichty 1970 | To describe the evidence which suggests the existence of a region within Colorado in which excess risks of occurrence of rheumatic fever were associated with socioeconomic factors manifest in 1959-61. | Ecologic | 75 cases RHD  Colorado, USA | Per cent of children aged 14-17 years in school  Median school years completed of persons aged 25 years of more | ARF cases and ARF/RHD death data from Colorado Department of Public Health | Varied by region. Average mean annual rate 14.0/100,000; range 7.6 to 64.6 /100,000 | **Possible association** Region with lowest proportion in school aged 14-17 (84.8% vs. population mean 89.4%) and lowest median school years completed (9.9% vs. population mean 11.7%) had highest ARF rate (64.6 vs. population average 14.0/100,000)  (no test of significance) |  | Poor: poor analysis, no multivariate. |
| Rosati et al. 1978 | To investigate the frequency of MS, rheumatoid arthritis, RHD and post-streptococcal nephritis in a population ethnically homogeneous and stable in size and composition, exclusively on the basis of differences in climatic and socioeconomic conditions. | Ecologic | 813 cases of RHD | Proportion of population who were illiterate | RHD diagnosed on hospital records of heart disease as a clinical manifestation of rheumatic fever diagnosed using revised Jones criteria | Various. 0.42 to 0.89/1,000 | **Possible association**  Regions with highest illiterate proportion had highest frequency of RHD:  Zone 1: 16%, 0.89/1000  Zone 2: 11%, 0.76/1000  Zone 3: 8%, 0.42/1000  Zone 4: 6%, 0.46/1000  (no test of significance) |  | Poor: ecologic unit too broad, no direct analysis. |

ARF: Acute rheumatic fever βHS: Beta haemolytic streptococci ECG: Electrocardiogram emmST: emm sequence type GAS Group A streptococci NA: Not applicable OR: odds ratio RHD: Rheumatic heart disease RR: Risk ratio USA: United States of America WHO: World Health Organization
