# Supplementary material for: The role of social determinants of health in the risk and prevention of group A streptococcal infection, acute rheumatic fever and rheumatic heart disease: A systematic review
Source: PLoS Negl Trop Dis. 2018 Jun 13;12(6):e0006577. doi: 10.1371/journal.pntd.0006577 (PMC6016946; doi:10.1371/journal.pntd.0006577)
Supplement: S5 Table — (DOCX) [file pntd.0006577.s008.docx]

**S5 Table. Summary of employment and GAS, ARF and RHD**

| Study details | Aim of study | Study design | Study population and setting | Measure of parental employment | Measure of outcome (GAS, ARF, RHD) | Outcome incidence/ prevalence | Results: univariate | Results: multivariate | Study quality |
| --- | --- | --- | --- | --- | --- | --- | --- | --- | --- |
| Adanja et al 1988 | To test the hypothesis regarding the influence of socioeconomic and some other factors on the occurrence of ARF. | Case control | 148 with first ARF attack, 444 controls matched for age, sex and place of residence (1:3)  Serbia | Parents employed: No-one/mother or father/both | ARF using revised Jones criteria | NA | **Positive association**  No-one/mother employed: 9.4% vs. 4.9%, Matched RR 10.37 (p=0.00014) |  | Poor to fair: cannot determine temporal association of exposure and outcome; no multivariate analysis. |
| Dobson et al 2012 | To investigate the role of environmental factors for RHD in Fiji. | Case control | 80 children aged 5-15 with RHD and 80 age and sex matched controls  Fiji | Mother not employed  Father not employed | Definite RHD diagnosed on echocardiogram using WHO criteria | NA | **Positive association**  Mother not employed 85% vs. 66% OR 2.6 (1.2-5.8)  **No association**  Father not employed |  | Poor: no power calculations, unstated number of controls from different source, participation rate 61%. |
| Grave 1957 | To investigate the factors of social and emotional forces in the aetiology of rheumatic fever. | Case control | 122 children aged between 2 and 12 with ARF, 100 controls from outpatient clinics within same age range  Sydney, Australia | Occupation of breadwinner:  Unskilled labourers  Receiving social service benefits or pension  Skilled or semi-skilled tradesman  Other occupations | ARF diagnosed on criteria of the Rheumatic fever council of the American Heart Association | NA | **Positive association** Unskilled labourers/receiving social services 30.7% vs. 11.5%, OR 3.42 (1.55-7.96)* |  | Poor: unmatched controls and no adjusting for differences, no power calculations, no test of significance |
| Hewitt & Stewart 1952 | This study deals with the social background of acute rheumatism. | Case control | 793 children aged 5-14 years  Sheffield, UK | Maternal employment  Housewives  Part-time  Full time  Father’s employment: long term unemployment | Acute rheumatism diagnosis based on notification criteria of the County Borough | NA | **Positive association** Maternal part-time and full time employment significantly more common in ARF group than controls (data not presented)  **No association**  Father’s long term unemployment |  | Poor: no baseline comparison, numbers not reported, participation rate not reported. |
| Riaz et al 2013 | To identify the risk factors of ARF and to explore the risk factors of RHD among ARF patients | Case control | 103 RHD cases, 103 ARF cases, 207 controls  Bangladesh | Occupation of mother: Housewife, working  Occupation of father: Labour intensive, moderate activity, sedentary activity. | ARF diagnosed using modified Jones criteria  RHD diagnosed by doppler echocardiography | NA | **Positive association** Mother working: No ARF 4.8%, ARF 11.7% OR 2.6 (1.1-6.2), RHD 15.5% OR 3.6 (1.6-8.3)    **No association**  Occupation of father |  | Fair: no matching, blinding of assessors not stated. |
| Okello et al 2012 | To investigate the role of socioeconomic and environmental factors in the pathogenesis of RHD in Ugandan patients. | Case control | 243 RHD cases, 243 controls aged between 5 and 60  Uganda | Study participant unemployed | RHD diagnosed using history ARF, clinical examination, echocardiogram criteria | NA | **Positive association**  Unemployed 78.9% vs. 54.7%, OR 3.09 (2.04-4.72) |  | Fair: unmatched controls, but randomly chosen and multivariate analysis performed, time period of participant selection not stated. |
| Vlajinac et al 1991 | To investigate the independent, unconfounded effect of risk factors for ARF identified in a previous study conducted on this population. | Case control | 148 with first ARF attack, 444 controls matched for age, sex and place of residence (1:3)  Serbia | Unemployment of parents | ARF diagnosed using revised Jones criteria | NA |  | **No association**  Unemployment of parents | Fair: temporal association of exposure and outcome not explicitly stated; |
| Eriksson et al 2013 | To examine the association between maternal size, and neonatal body size and placental size and the occurrence of chronic rheumatic heart disease within the Helsinki Birth Cohort | Cohort | 101 RHD cases  Helsinki, Finland | Father’s occupational status on birth certificate | RHD cases ascertained from centralised national hospital admission and death database using ICD codes. |  | **No association**  Father’s occupational status |  | Fair: no multivariate analysis, no confounders discussed, no power calculation. |
| Mirabel et al 2015 | To address the outcomes and modalities of RHD screening through a cohort of children with and without RHD who took part in the first large RHD echocardiography based surveillance programme. | Cohort | 114 cases of RHD from cohort of 157 and 227 controls selected randomly from classmates, matched for ethnicity and classroom  New Caledonia | Maternal employment: No formal occupation, receives salary. | Persistence of RHD diagnosed using World Heart Foundation criteria.    ARF diagnosed using Australian Guideline for Prevention, Diagnosis and Management of ARF and RHD. | RHD: 890/ 100,000  ARF: 10.28/1,000/year | **No association**  Maternal employment |  | Fair: no power calculation, >20% lost to follow up. |
| Westerling et al 1996 | To analyse socioeconomic differences in avoidable mortality in Sweden and to discuss related methodological issues. | Cohort | 4,239,283 Swedish people aged 21-64 in 1986.  Sweden | Case employed, non-employed. | RHD mortality ascertained from national cause of death register 1986-1990. | 28 deaths from RHD | **Positive association**  SMR 56 employed vs. 293 non-employed |  | Fair: good quality data sources and design, small case numbers. |
| Bassili et al 2000 | To evaluate the current regimen of secondary prophylaxis available to disadvantaged Egyptian children suffering from RHD who were attending various children’s hospitals in Alexandria. | Cross section | 150 children with RHD | Paternal occupation: Technical/professional, unskilled worker, unemployed. | Diagnosis of RHD based on a documented history of ARF with characteristic murmur confirmed by Doppler echocardiography | NA | **No association**  Paternal occupation |  | Poor to fair: no power calculation, limited factors included |
| Ledos et al 2015 | To conduct an echocardiographic screening study to evaluate the prevalence of RHD among young adults from New Caledonia | Cross section | 834 subjects aged 18-22 years | Occupation of study participant: Students, apprentices, prisoners | RHD diagnosis made using screening with echocardiography and interpretation by three cardiologists according to pre-defined protocol. | 5.9/1,000 | **No association**  Occupation of study participant |  | Poor to fair: well designed but underpowered |
| Lumsden et al 2016 | To describe the clinical profile of RHD patients, identify demographic factors associated with RHD and determine if a pattern of geographic distribution of RHD exists throughout the region. | Cross section | 906 patients under 50 years of age | Occupation of study participant: Employed, self-employed, unemployed, student, housewife. | Diagnosis of RHD using hospital record diagnoses confirmed by cardiologist and standardised echocardiography report. |  | **Positive association**  Employed 3.4% vs. 10.2%, self-employed 9.8% vs. 12.7%, unemployed 3.4% vs. 3.1%, student 18.7% vs. 9.3%, housewife 11.7% vs. 10.2% (p<0.001)  (Not recorded 52.9% vs. 54.6%) |  | Fair: well described study, limited multivariate analysis of factors. |
| Morton & Lichty 1970 | To describe the evidence which suggests the existence of a region within Colorado in which excess risks of occurrence of rheumatic fever were associated with socioeconomic factors manifest in 1959-61. | Ecologic | 75 cases RHD  Colorado, USA | Ratio of non-workers to workers at regional level | ARF cases and ARF/RHD death data taken from Colorado Department of Public Health | Varied by region. Average mean annual rate 14.0/100,000; range 7.6 to 64.6 /100,000 | **Possible association**  Region with highest ratio of non-workers to workers (2.23 vs. population mean 1.60) had highest ARF incidence (64.6 vs. population average 14.0/100,000)  (no test of significance) |  | Poor: poor analysis, no multivariate. |
| Rosati et al. 1978 | To investigate the frequency of MS, rheumatoid arthritis, RHD and post-streptococcal nephritis in a population ethnically homogeneous and stable in size and composition, exclusively on the basis of differences in climatic and socioeconomic conditions. | Ecologic | 813 cases of RHD | Proportion of population working | RHD diagnosed on hospital records of heart disease as a clinical manifestation of rheumatic fever diagnosed using revised Jones criteria | Various. 0.42 to 0.89/ 1000 | **Possible association**  Regions with lowest % working population had highest frequency of RHD:  Zone 1: 21%, 0.89/1000  Zone 2: 23%, 0.76/1000  Zone 3: 28%, 0.42/1000  Zone 4: 33%, 0.46/1000  (no test of significance) |  | Poor: ecologic unit too broad, no direct analysis. |

*Test of significance calculated for systematic review from original study data

ARF: Acute rheumatic fever ICD: International Classification of Diseases GAS Group A streptococci NA: Not applicable OR: odds ratio RHD: Rheumatic heart disease RR: Risk ratio SMR: Standardised mortality ratio UK: United Kingdom USA: United States of America WHO: World Health Organization
