# Supplementary material for: The role of social determinants of health in the risk and prevention of group A streptococcal infection, acute rheumatic fever and rheumatic heart disease: A systematic review
Source: PLoS Negl Trop Dis. 2018 Jun 13;12(6):e0006577. doi: 10.1371/journal.pntd.0006577 (PMC6016946; doi:10.1371/journal.pntd.0006577)
Supplement: S6 Table — (DOCX) [file pntd.0006577.s009.docx]

S6 Table. Summary of income and GAS infection, ARF and RHD

| Study details | Aim of study | Study design | Study population and setting | Income measure and strata | Measure of outcome (GAS, ARF, RHD) | Outcome incidence/ prevalence | Results: univariate | Results: multivariate | Study quality |
| --- | --- | --- | --- | --- | --- | --- | --- | --- | --- |
| Adanja et al 1988 | To test the hypothesis regarding the influence of socioeconomic and some other factors on occurrence of ARF. | Case control | 148 with first ARF attack and 444 controls matched for age, sex and place of residence (1:3)  Serbia | Family income (in Dinar): <3000, 3001-5999, 6000-8999, 9000+ | ARF using revised Jones criteria | NA | **No association**  Family income |  | Poor to fair: temporal association of exposure and outcome not explicitly stated; no multivariate analysis. |
| Grave 1957 | To investigate the effect of social and emotional factors in the aetiology of rheumatic fever in a group of children. | Case control | 122 children aged 2-12 years with acute rheumatic fever and 100 controls  Sydney, Australia | Adequate, marginal or inadequate family income 2 years prior to illness  Adequate, marginal or inadequate family income at time of illness  Haphazard or unsatisfactory management of family income | ARF diagnosed on criteria of the Rheumatic fever council of the American Heart Association | NA | **Positive association**  Marginal or inadequate income 2 years prior 56% vs. 34%, OR 2.45 (1.36-4.41)  Marginal or inadequate income at time of illness 53% vs. 31%, OR 2.54 (1.41-4.61)  Haphazard/unsatisfactory management 42% vs. 13%, OR 4.78 (2.32-10.31)* |  | Poor: unmatched controls and no adjusting for differences, no power calculations, no test of significance given |
| Gray et al 1952 | To follow up a previous study after a long period of observation of families to compare further the rheumatic and control families with respect to hereditary and certain environmental factors which might be responsible for the familial prevalence of rheumatic fever. | Case control | 40 families with a ARF case and 30 control families with a scarlet fever case  Connecticut, USA | Average family income | ARF diagnosed by physicians at time of acute episode (criteria not given).  RHD diagnosed on clinical criteria of the New York and American Heart Association | NA | **No association**  Average family income |  | Poor: high proportion of cases lost to follow up, test of significance not reported |
| Hewitt & Stewart 1952 | To study the social background of notified ARF cases. | Case control | 793 children aged 5-14 years  Sheffield, UK | Family income per head: Low ≤17s 11d, Medium 18s-32s 11d, High ≥33s | Formal notification of ARF, confirmed by consultant according to “common standard” | NA | **Positive association**  Low income 22.9% vs. 14.9%  High 20.1% vs. 28.3%  χ^2^≥10.44 (p<0.01) |  | Poor: no baseline comparison, numbers not reported, participation rate not reported. |
| Riaz et al 2013 | To identify the risk factors of ARF and to explore the risk factors for developing RHD among ARF patients | Case control | 103 RHD, 103 ARF cases, and 207 controls  Bangladesh | Monthly family income >10,000 Tk/month | ARF based on modified Jones criteria  RHD diagnosed by doppler echocardiography | NA | **No association**  Monthly family income |  | Fair: no matching, blinding of assessors not stated. |
| Kurahara et al 2006 | To determine factors in prevalence rates of ARF in a multiethnic population. | Case control | 26 ARF cases and 41 controls with other heart condition (all on Medicaid)  Hawaii, USA | Average monthly household income <$25,000/year | ARF diagnosed using modified Jones criteria | NA | **No association**  Monthly family income |  | Fair: no matching, small sample size |
| Okello et al 2012 | To investigate the role of socioeconomic and environmental factors in the pathogenesis of RHD in Ugandan patients. | Case control | 243 RHD cases and 243 controls aged 5-60 years  Uganda | Monthly income (USD) <25, 25-49.5, 50-99.5, ≥100. | RHD diagnosed using history ARF, clinical examination, echocardiogram criteria | NA | **Positive association** Income ≥100 6.7% vs. 35.8%, OR 14.7 (5.9-36.1) | **No association** Monthly income | Fair: unmatched controls, but randomly chosen and multivariate analysis performed, time period of participant selection not stated. |
| Zaman et al 1997 | To examine data on patients with proven GAS infection presenting to a ARF hospital to identify socio-economic factors that may need further exploration. | Case control | 44 ARF cases and 86 controls aged 5-20 years with recent GAS infection  Dhaka, Bangladesh | Mean family income: taka/month/person  Low Income (USD$): ≤10.71 /month | ARF diagnosed on revised Jones criteria | NA | **Positive association** Family income 600 vs. 750 (p=0.02)  Low income 37% vs. 20% (p=0.04) | **No association** Family income, low income | Fair to good: no power calculations |
| Zaman et al 1998 | To explore further the nutritional factors that may be associated with ARF. | Case control | 60 ARF cases and 104 controls aged 5-20 years with recent GAS infection  Dhaka, Bangladesh | Log income (taka/month) | ARF diagnosed using updated Jones criteria | NA | **Positive association**  Log income 8.1 vs. 8.4 (p=0.002) |  | Fair to good: no power calculations. |
| Meira et al 2005 | To study the progress of valvar disease by means of clinical and echocardiographic evaluations and to identify the independent variables that predict severe chronic valvar disease. | Case series | 258 children and adolescents diagnosed with ARF between 1983 to 1998.  Belo Horizonte, Brazil | Family income ≤3 X minimum wage | ARF diagnosed using revised Jones criteria |  | **No association**  Family income | **No association** Family income | Fair: reasonable breadth of factors, multivariate analysis, no power calculation |
| Westlake et al 1990 | To assess the current incidence of disease, to characterise its epidemiology, to assess the antecedent symptoms and treatment of such and to describe the clinical manifestations of ARF in these patients. | Case series | 26 cases ARF aged 4 to 14  Tennessee, USA | Below poverty line (<$US 11,612) | ARF diagnosed using revised Jones criteria |  | **Possible association**  Below poverty line ARF cases 19% vs. general population 9.2%  (no test of significance) |  | Poor: no statistical tests undertaken, small sample. |
| Jackson et al 1947 | To determine the recurrence rate of a group of rheumatic subjects who received special attention to improve their diets and level of environmental care and evaluate the relation of environmental factors to the course of the disease. | Cohort | 266 children with history of ARF under the Supervision of the University hospital and state children’s mobile clinics.  Iowa, US | Economic condition of the home:  Good (sufficient income for diet, clothing shelter)  Fair (marginal income for diet, clothing shelter)  Poor (not sufficient for diet, clothing shelter) | ARF and recurrence of ARF diagnosed using Jones criteria.  Inactive disease diagnosed using Taran criteria |  | **No association**  Home economic situation and recurrence of ARF |  | Poor: no adjustment, uneven follow up. |
| Tay et al 1981 | To collect information pertaining to the following points:  the carriership of beta-haemolytic streptococcal throat and skin diseases; the incidence of various serological groups of haemolytic streptococci and of the types of group A streptococci; the role played by the various factors like race, season of the year, socio-economic status etc. on the clinical, epidemiological and microbiological patterns of the streptococcal carriership and of the disease; the assessment of the best therapy regime for streptococcal disease applicable in the local conditions | Cohort | 491 primary school students  Singapore | Family monthly income ($Singapore) <500, 500-999, 1,000-1,499, >1,500. | Throat and skin swab cultures | Overall cumulative incidence of GAS throat carrier rate was 46.7% | **Positive association** GAS cumulative incidence <$500 62% vs. 38%, ≥$500, RR 2.70 (2.03-3.58) * |  | Poor to fair: >20% attrition, no power calculation, poor reporting of statistics. |
| Ba-Siddik et al 2011 | To estimate the prevalence of RHD among school-children aged 5-16 years in Aden (Yemen). | Cross section | 6000 school children aged 5-16 years  Aden, Yemen | Family income (Yemeni riyals): low (<5000), intermediate (5000-10,000), high (>10000) | Clinical examination with echocardiography if murmur present. Definitive RHD diagnosed on modified Duckett Jones’ criteria and WHO criteria for Doppler abnormalities. | 36.5/1000 children (95% CI 32-41.6). | **Positive association** Proportion of low income RHD 49.3% vs. non-RHD 21.6% (p<0.001) |  | Poor to fair: no adjustment, non-blinded assessors |
| Hammon et al 1950 | A preliminary study to explore the usefulness of a test to indicate past infection with poliomyelitis and the disease’s possible correlation to streptococcal infections. | Cross section | 653 children aged 1-15  California, USA | Economic group of family: low, high | ASOT | Range of 11.1% to 100% ASOT positive | **Possible association** Greater proportion of children with positive ASOT from low income families compared to high income (graphed data only) |  | Poor: inadequate description of methods. |
| Likitnukal et al 1994 | To evaluate the factors influencing the colonization of streptococci in school age children. | Cross section | 1547 school children aged 6-11 years  Bangkok, Thailand | Low family income ≤$160 US/month | Throat swab culture for BHS and GAS | GAS 18%  βHS 1747 47% | **Positive association** Proportion with low family income GAS positive 61.9% vs. GAS negative 52.1%, RR 1.49 (1.18-1.90)  **No association**  Low family income and BHS |  | Poor: high attrition, no power calculation. |
| Vashistha et al 1993 | To find out the magnitude of the problem of RHD in children and to make an early diagnosis to prevent further complications. | Cross section | 8,449 school children aged 5-15 years  Agra, India | Monthly family income (Rupees): Economically backward ( <700Rs), Low income( 700- 1500Rs), Middle income (1500-2500 Rs), High income (>2500Rs) | ARF diagnosed using revised Jones criteria.  RHD diagnosed on clinical, radiography, ECG and echocardiographic findings | RHD 1.4/1000 | **No association**  Monthly family income |  | Poor: poor reporting of results and statistical analysis. |
| Jaine et al 2011 | To test the hypothesis that household crowding was positively associated with ARF incidence and whether there was a dose-response relationship between the exposure and ARF risk. | Ecologic | 1,249 ARF cases between 1996 and 2005.  New Zealand | Mean household income level of the census area unit | ARF diagnosed from hospital recorded diagnosis | Average annual rate ARF 3.4/ 100,000 | **Positive association** ARF rate ratio by income quintile   1. 1.0 (Reference) 2. 1.7 (1.1-2.5) 3. 2.8 (2.0-4.1) 4. 5.0 (3.6-7.1) 5. 17.5 (12.7-24.3) | **Positive association** Median household income IRR 0.985 (0.974-0.998) | Good: well described study, consistent and valid exposure and outcome measures. |
| Lue et al 1979 | To collect information on streptococcal infections, prevalence and severity of ARF and RHD and their long term follow up. | Ecologic | Various | Per capita income nationally in US$ | GAS diagnosed on throat swab culture  Diagnosis of ARF was made based on the revised or modified Jones criteria. | Range for GAS 1.6-29.5%  RHD 0.3 to 2.7/1000 | **No association**  GAS prevalence & per capita income  RHD prevalence & per capita income |  | Poor: heterogeneity in methods, populations and results. Ill-defined ecologic units. |
| Morton & Lichty 1970 | To describe the evidence which suggests the existence of a region within Colorado in which excess risks of occurrence of rheumatic fever were associated with socioeconomic factors manifest in 1959-61. | Ecologic | 75 cases RHD  Colorado, USA | Mean income  %< $3,000  %>$10,000 | ARF cases and ARF/RHD death data taken from Colorado Department of Public Health | Varied by region. Average mean annual rate 14.0/100,000; range 7.6 to 64.6 /100,000 | **Possible association**  Region with lowest mean income ($2,486 vs. population mean $3,671), highest proportion <$3000 (40.6% vs. population mean 18.3%) and lowest proportion >$10,000 (7.1 vs. population mean 14.6) had highest ARF rate (64.6 vs. population average 14.0/100,000) (No test of significance) |  | Poor: poor analysis, no multivariate. |
| Odio et al 1986 | To undertake a 10 year study of ARF in the San Fernando Valley | Ecologic | 21 cases ARF  Los Angeles, USA | Average income <$18,000 by community | ARF diagnosed using revised Jones criteria | 0.21/100,000 | **No association**  Average income <$18,000 in ARF vs. population |  | Poor: small study, limited factors, not well described. |

*Test of significance calculated for systematic review from original study data

ARF: Acute rheumatic fever ASOT: Anti-streptolysin O titre βHS: Beta haemolytic streptococci ECG: Electrocardiogram HR: Hazard ratio IRR: Incidence rate ratio GAS Group A streptococci NA: Not applicable OR: odds ratio RHD: Rheumatic heart disease RR: Risk ratio UK: United Kingdom USA: United States of America WHO: World Health Organization
