# Supplementary material for: The role of social determinants of health in the risk and prevention of group A streptococcal infection, acute rheumatic fever and rheumatic heart disease: A systematic review
Source: PLoS Negl Trop Dis. 2018 Jun 13;12(6):e0006577. doi: 10.1371/journal.pntd.0006577 (PMC6016946; doi:10.1371/journal.pntd.0006577)
Supplement: S7 Table — (DOCX) [file pntd.0006577.s010.docx]

S Table 7. Summary of nutrition and GAS infection, ARF and RHD.

| Study details | Aim of study | Study design | Study population and setting | Measure of nutrition | Measure of outcome (GAS, ARF, RHD) | Outcome incidence/ prevalence | Results univariate | Results multivariate | Study quality |
| --- | --- | --- | --- | --- | --- | --- | --- | --- | --- |
| Adanja et al 1991 | To retest the hypothesis of an association between diet and the occurrence of ARF. | Case control | 148 with first ARF attack and 444 controls matched for age, sex and place of residence (1:3)  Serbia | Consumption of certain foods: cheese, milk eggs (daily); meat, vegetables, fruit (< 3 times week).  Underweight= Bodyweight >10% below normal using Baldwin-Wood’s tables  Overweight= Bodyweight >10% over normal using Baldwin-Wood’s tables | ARF using revised Jones criteria  Frequent sore throat=> 1/year | NA | **Positive association**  Underweight 25.0% vs. 14.6%, OR 2.23 (1.09-4.53)  **No association**  Milk, cheese, eggs, meat, vegetables, fruit. | **Positive association**  Underweight OR 1.39 (1.07-1.82)  Without sore throat only:  No eggs OR 2.08 (1.10-3.93) | Poor: Results not presented in entirety. Temporal association of exposure and outcome not explicitly stated. |
| Vlajinac et al 1991 | To investigate the independent, unconfounded effect of risk factors for ARF identified in a previous study conducted on this population. | Case control | 148 with first ARF attack and 444 controls matched for age, sex and place of residence (1:3)  Serbia | Underweight= Bodyweight >10% below normal using Baldwin-Wood’s tables | ARF diagnosed using revised Jones criteria | NA |  | **Positive association**  Underweight RR 1.42 (1.08-1.86) | Fair: temporal association of exposure and outcome not explicitly stated. |
| Vlajinac et al 1989 | To test the hypothesis that socio-economic factors are related to rheumatic fever and make an additional comparison for those with and without a history of sore throat. | Case control | 148 with first ARF attack and 444 controls matched for age, sex and place of residence (1:3)  Serbia | Undernourishment= Bodyweight >10% below normal using Baldwin-Wood’s tables | ARF diagnosed using revised Jones criteria  Frequent sore throat= >1/year | NA | **Positive association**  Participants without frequent sore throat:  Undernourishment RR 2.69 (1.41-5.12)  **No association**  Participants with frequent sore throat:  Undernourishment |  | Poor to fair: temporal association of exposure and outcome not explicitly stated; stratified only by sore throat frequency. |
| Wilcox & Galloway 1954 | To determine the relationship between diet and physical well-being of children with and without a history of rheumatic fever. | Case control | 131 children with ARF aged 5-19 years and matched controls  Utah, USA | Average daily intake of nutrients derived from 7 day dietary recalls. Included: Energy (calories), protein, fat, calcium, iron, vitamin A, thiamine, riboflavin, niacin, ascorbic acid.  Caloric need met  Height and weight compared to Baldwin & Wood standard.  Height and weight compared to Stuart and Meredith standard | ARF diagnosis based on history of ARF reported in records of Ogden Rheumatic Fever Clinic | NA | **Positive association**  Girls all ages:  Energy, protein, far, iron, thiamine, niacin (data not presented, p<0.05)  10-12 year old girls:  Energy 2198 calories vs. 2415 calories (p<0.05)  Fat 92.7gm. vs. 0.53gm. (p<0.05)  <10 year old boys:  Thiamine 1.22mg vs. 1.05mg (p<0.05)  Anthropometrics:  >10 % Underweight 30% vs. 23% (p<0.05)  **No association**  Nutrient intakes for all other groups, height and weight compared to Stuart and Meredith standard, Caloric need met |  | Poor: no case definition given, no baseline comparisons between case/controls, no sample size calculation. |
| Zaman et al 1997 | To examine data on patients with proven GAS infection presenting to a ARF hospital to identify socio-economic factors that may need further exploration. | Case control | 44 ARF cases and 86 controls aged 5-20 years with recent GAS infection  Dhaka, Bangladesh | Weight for age <80%  Height for age <90%  Weight for height <80%  Arm circumference for age <80% | ARF diagnosed on revised Jones criteria | NA | **Positive association**  Height for age <90% 32% vs. 28% (p=0.02)  **No association**  Weight for age, weight for height, arm circumference |  | Fair to good: no power calculations |
| Zaman et al 1998 | To examine whether ARF is associated with serum protein concentrations and body iron stores in Bangladeshi children. | Case control | 44 ARF cases, 44 age and sex matched controls | Total protein (g/l)  Serum albumin concentrations (g/l)  Cholesterol mmol/l (Total, HDL LDL)  Triglycerides (mmol/l)  Haemoglobin  Iron (µmol /l)  Total iron binding capacity (µmol/l)  Transferrin saturation (%) | ARF diagnosed using updated Jones criteria | NA | **Positive association**  Albumin 44 vs. 48 OR 0.72 (0.59-0.88)  HDL 0.91 vs. 1.06 OR 0.05 (0.01-0.49)  Haemoglobin 115 vs. 123 OR 0.94 (0.90-0.99)  Packed cell volume 37 vs. 39.3 OR 0.73 (0.59-0.91)  Iron 9 vs. 14 OR 0.81 (0.71-0.93)  Transferrin saturation 15.8 vs. 22.8 OR 0.89 (0.83-0.96)  **No association**  Total protein, Total cholesterol, LDL, triglycerides, total iron binding capacity | **Positive association**  Mean albumin OR 0.75 (0.6-0.95)  Packed cell volume OR 0.69 (0.50-0.97)  Iron OR 0.82 (0.68-0.97)  Transferrin saturation OR 0.90 (0.92-0.98) | Fair to good: no power calculations |
| Zaman et al 1998 | To explore further the nutritional factors that may be associated with ARF. | Case control | 60 ARF cases, 104 controls aged 5-20 years with recent GAS infection  Dhaka, Bangladesh | Weight for age <80%  Height for age <90%  Weight for height <80%  Arm circumference for age <80%  Triceps skinfold thickness for age <80%  Food frequency questionnaire to determine look intake of: egg, milk, beef, mutton, chicken, fish, pulses, fruits, *paratta*, *ruti*, leafy vegetables, other vegetables  Use of soybean cooking oil | ARF diagnosed using updated Jones criteria | NA | **Positive association**  Height for age<90% 36% vs. 13%, OR 3.83 (1.74-8.40)  Weight for age<80% 83% vs. 67%, OR 2.41(1.12-5.57)  Upper arm circumference <80% 76% vs. 46%, OR 3.76 (1.87-7.89)  Low intake of food items:  Egg 68% vs. 36% OR 3.81 (1.95-7.63)  Milk 61% vs. 37% OR 2.60 (1.36-5.08)  Chicken 67% vs. 43% OR 2.62 (1.35-5.21)  Pulses 61% vs. 44% OR 1.98 (1.03-3.84)  Fruits 59% vs. 39% OR 2.29 (1.20-4.45)  *Ruti* 65% vs. 37% OR 3.15 (1.61-6.34)  Soybean cooking oil 66% vs. 87% OR 0.28 (0.12-0.62)  **No association**  Weight for height  Triceps skinfold thickness for age<80%  Beef, mutton, fish, *paratta*, leafy vegetables, other vegetables. | **Positive association**  Upper arm circumference for age <80%, OR 2.40 (1.04-5.77)  Low intake of food items:  Egg OR 2.29 (1.01-5.27) | Fair to good: no power calculations. |
| Meira et al 2005 | To study the progress of valvar disease by means of clinical and echocardiographic evaluations and to identify the independent variables that predict severe chronic valvar disease. | Case series | 258 children and adolescents diagnosed with ARF between 1983 to 1998.  Belo Horizonte, Brazil | Weight at registration <10 centile | ARF diagnosed using revised Jones criteria | NA | **No association**  Weight <10 centile | **No association** | Fair: reasonable breadth of factors, multivariate analysis, no power calculation |
| Jackson et al 1947 | To determine the recurrence rate of a group of rheumatic subjects who received special attention to improve their diets and level of environmental care and evaluate the relation of environmental factors to the course of the disease. | Cohort | 266 children with history of ARF under the Supervision of the University hospital and state children’s mobile clinics.  Iowa, USA | Classified into groups according to way they followed prescribed diet  Good: dietary advice adhered to at all time  Fair: Dietary advice followed most of time  Fair to good: fluctuations from fair to good  Poor: Diet definitely low in one or more essential food  Poor to fair: Fluctuating from poor to fair  Very poor: Diet very inadequate. | ARF and recurrence of ARF diagnosed using Jones criteria.  Inactive disease diagnosed using Taran criteria |  | **Positive association**  Diet pattern in recurrence vs. non recurrence:  Good 14.6% vs. 37.9%  Fair to good 12.5% vs 16.3%  Fair 37.5% vs. 35.0%  Poor to fair 20.8% vs. 5.4%  Poor 14.6% 5.4%  χ^2^=18.45 |  | Poor: no adjustment, uneven follow up. |
| Coburn 1960 | To study the effect of diet and poverty on the rheumatic process by assessing diets of poor children without ARF, poor children with ARF, and economically moderate children without ARF. | Cross section | 1039 children aged 7-15 years  Chicago, USA | Diet recall questionnaire to determine mean consumption of: eggs (per week), milk (cups per day), total protein (gm per day), vitamin A, vitamin C. | Children diagnosed with ARF and who attended cardiac-school classes | NA | **Positive association**  Mean egg consumption: ARF children 2.8, non-ARF children in poverty 3.5, non-ARF non-poverty 4.3 (p<0.01)  **No association**  Milk, Total protein, Vitamin A, Vitamin C |  | Poor: poor description of methods, case ascertainment, no analysis of confounders. |
| Longo-Mbenza et al 1998 | A study of the prevalence of RHD in children of Kinshasa using echocardiography to confirm the diagnosis and to verify the possible relationship between host, slum environment and RHD. | Cross section | 4848 students aged 5-16 years  Kinshasa, Democratic Republic of Congo | BMI < mean-1 SD | RHD diagnosed on echocardiography of suspect cases. | 14.03/1,000 |  | **Positive association**  Low BMI OR 2.68 (1.43-5.01) | Fair: limited results presented. |
| Poppi et al 1953 | To ascertain the prevalence of ARF and RHD in a region of Italy where the high incidence of both complaints is well known to practicing physicians, and to evaluate the weight of some factors generally admitted as important in the pathogenesis of the disease. | Cross section | 930 female manual labourers aged 14-70 years  Po valley, Italy | Dietary intake poor, normal or rich for: Carbohydrates, Proteins, Fat, Fresh fruit and vegetable.  Dark adaptation (proxy for vitamin A) | ARF based on clinical history of acute migrating polyarthritis, confining patient to bed with fever and subsiding after salicylates, or of Sydenham’s chorea.  RHD diagnosed with examination, orthodiagram of the heart and ECG. | 14.9% history of ARF or chorea  8.7% RHD | **Positive association**  Correlation between poor protein intake and ARF (χ^2^=45.4, P<0.005)  **No association**  Carbohydrates, fat, fresh fruit and vegetables. |  | Poor: ill-defined exposure and poor presentation of results. |
| Saxena et al 2011 | To estimate the prevalence of clinical and subclinical RHD, to identify risk factors associated with RHD and to study the natural history of children with echocardiographically detected RHD. | Cross section | 6,270 school children aged 5-15 years  Rural area,  North India | BMI  Waste circumference (cm)  Height (cm)  Weight (kg) | RHD diagnosed using modified WHO criteria of echocardiogram. | 20.4/1,000 | **No association**  BMI, waist circumference, height, weight. |  | Fair: no power calculation. |
| Steer et al 2009 | To determine the prevalence of RHD in Fiji, by using a three-stage screening protocol that included auscultation as the first screening step. | Cross section | 3,462 children aged 5-15 years  Fiji | Weight for age  Height for age  BMI | RHD diagnosed on 3 step screening: auscultation, limited echocardiography, full echocardiography using WHO/USA NIH 2005 consensus case definition. | 4.1/1,000 | **No association**  Weight for age  Height for age  BMI |  | Fair: no sample size estimate for exposure factors |
| Roberts et al 2015 | To describe the prevalence of definite and borderline RHD in Indigenous children in different regions of Australia and the Torres Strait and to inform decision making about the potential impact and usefulness of echocardiographic screening for RHD in different Australian regions. | Ecologic | 3,964 Indigenous children aged 5-14 years in four different regions  Australia | Weight (kgs)  Height (cm)  BMI | RHD diagnosed using World Heart Foundation echocardiographic criteria for all participants. | Various. From 4.7/1000 in Far North Queensland to 15/ 1000 in Top End region. | **Possible association**  Anthropometric measures lower in the Top End community compared to other 3 communities which followed RHD prevalence (15.0/1000 vs. 6.7, 4.7, 8.9/1000)  Weight 26.5 vs 29.8, 28.5, 27.4 (p<0.001)  Height 133.0 vs. 135.0, 133.4, 133.0 (p=0.01), BMI 15.1 vs. 16.2, 16.2, 15.7 (p<0.001) (no direct test of association) |  | Poor to fair: low participation rate, no test of significance of SES factors and RHD prevalence. |

*Test of significance calculated for systematic review from original study data

ARF: Acute rheumatic fever BMI: Body Mass Index ECG: Electrocardiogram GAS Group A streptococci HDL High density lipoprotein LDL: Low density lipoprotein NA: Not applicable NIH: National Institute of Health OR: odds ratio RHD: Rheumatic heart disease RR: Risk ratio USA: United States of America WHO: World Health Organization
