# Supplementary material for: The role of social determinants of health in the risk and prevention of group A streptococcal infection, acute rheumatic fever and rheumatic heart disease: A systematic review
Source: PLoS Negl Trop Dis. 2018 Jun 13;12(6):e0006577. doi: 10.1371/journal.pntd.0006577 (PMC6016946; doi:10.1371/journal.pntd.0006577)
Supplement: S8 Table — (DOCX) [file pntd.0006577.s011.docx]

S Table 8. Summary of socioeconomic status and GAS infection, ARF and RHD

| Study details | Aim of study | Study design | Study population and setting | Measure of socioeconomic status | Measure of outcome (GAS, ARF, RHD) | Outcome incidence/ prevalence | Results: univariate | Results: multivariate | Study quality |
| --- | --- | --- | --- | --- | --- | --- | --- | --- | --- |
| Dobson et al 2012 | To investigate the role of environmental factors for RHD in Fiji. | Case control | 80 children aged 5-15 with RHD and 80 age and sex matched controls  Fiji | Usual mode of transport car, no car | Definite RHD diagnosed on echocardiogram using WHO criteria | NA | **No association** Ownership of car |  | Poor: no power calculations, unstated number of controls from different source, participation rate 61%. |
| Grave 1957 | To investigate the factors of social and emotional forces in the aetiology of rheumatic fever. | Case control | 122 children aged 2-12 with ARF, 100 controls from outpatient clinics within same age range  Sydney, Australia | Maternal efficiency composite score of 1-3 for each of: appearance of furniture, cleanliness of house, appearance of mother | ARF diagnosed on criteria of the Rheumatic fever council of the American Heart Association | NA | **Positive association**  Low maternal efficiency 20.7% cases vs. 5.0% controls, OR 5.05 (1.77-17.51)* |  | Poor: unmatched controls and no adjusting for differences, no power calculations, no test of significance |
| Hewitt & Stewart 1952 | This study deals with the social background of acute rheumatism. | Case control | 793 children aged 5-14 years  Sheffield, UK | Social class I-V based on father’s occupation (1=highest) | Acute rheumatism diagnosis based on notification criteria of the County Borough | NA | **Positive association**  Ratio of actual to expected ARF cases  I&II 48, III 89, IV 125, V 147 (p=<0.01) |  | Poor: no baseline comparison, numbers not reported, participation rate not reported. |
| Kurahara et al 2006 | To determine factors in prevalence rates of ARF in a multiethnic population. | Case control | 26 cases ARF, 41 controls with other heart condition (all on Medicaid)  Hawaii, USA | Ownership of house | ARF diagnosed using modified Jones criteria | NA | **No association**  Ownership of house |  | Fair: no matching, small sample size |
| Brownell & Bailen-Rose 1973 | To study children in the Manhattan borough of New York City with initial or recurrent ARF attacks in 1963-65 over a period of time. | Case series | 324 ARF cases in children aged 5-14  New York, USA | Social class of district; not defined. | Program cardiologist confirms a reported diagnosis of ARF | Average annual incidence 61/100,000 | **Possible association**  Highest attack rates 78 to 79/100,000 were in the two districts of Pueto Rican residents living in most congested “ghetto” areas (no test of significance) |  | Very poor: Poorly described methods and results. |
| Diamond 1957 | To evaluate the relative roles of unfavourable environmental factors and hereditary susceptibility using the patients admitted to La Rabida Sanitarium for ARF. | Case series | 1205 cases ARF  Chicago, USA | Families of cases allocated to environmental groups I-IV based on ‘Wilson and Schweitzer’ composite indicator of adequacy of housing space and heating, family budget, food, and clothing (I=highest). | ARF diagnosed using original hospital admission records | NA | **Positive association**  Incidence of ARF among siblings in group IV families once index case excluded 59/207 vs. combined group I-III 207/891 (p<0.05) |  | Poor: limited analysis, no description of case ascertainment. |
| Veasy et al 1994 | To follow up a study of a resurgence of ARF in the intermountain area surrounding Salt Lake City, to determine whether the socioeconomic status of the patient population and the clinical presentation had changed since the initial report and to assess in patients with pure chorea and isolated rheumatic polyarthritis the incidence of silent mitral regurgitation by echocardiographic evaluation. | Case series | 274 cases ARF aged 2 to 17  Utah, USA | Low, middle, upper class.  Medical insurance coverage | Diagnosed ARF using revised Jones criteria | NA | **No association**  Class, insurance coverage |  | Poor: limited factors included and poor display or results. |
| Westlake et al 1990 | To assess the current incidence of ARF, to characterise its epidemiology, to assess the antecedent symptoms and treatment of such and to describe the clinical manifestations of ARF in these patients. | Case series | 26 cases ARF aged 4 to 14  Tennessee, USA | Socioeconomic status ranked 1-5 based on Hollingshead criteria- two factor index of household head education and occupation (1=highest). | ARF diagnosed using revised Jones criteria | NA | **Possible association**  SES Hollingshead level 5 44% ARF cases vs. vs. 25% general population  (no test of significance) |  | Poor: no statistical tests undertaken, small sample. |
| Jackson et al 1947 | To determine the recurrence rate of a group of rheumatic subjects who had received special attention to improve their diets and level of environmental care and evaluate the relation of environmental factors to the course of the disease. | Cohort | 266 children with history of ARF under the Supervision of the University hospital and state children’s mobile clinics.  Iowa, USA | 3 strata of home social situation:  Good: parents emotionally stable, sincere in their efforts, cooperated with doctor.  Fair: level of care was marginal due to conditions which could not be controlled and where co-operation was not always constant  Poor: parents were ignorant or indifferent, poor parental relationship, home life disrupted, no help obtained from community. | ARF and recurrence of ARF diagnosed using Jones criteria.  Inactive disease diagnosed using Taran criteria | NA | **No association**  Home social situation and recurrences |  | Poor: no adjustment, uneven follow up. |
| Nandi et al 2001 | To estimate the incidence and risk factors for GAS sore throat among school-aged children in northern India | Cohort | 536 children aged 5-15 in 26 in peri-urban slum households  Chandigarh, India | Socioeconomic status:  Lower, lower-middle, upper middle. | Throat swab culture for GAS | 1 episode/child/year among 5-15 year olds. | **No association**  Socioeconomic status |  | Poor to fair: no power calculations, no multivariate analysis. |
| Quinn et al 1978 | To investigate the incidence of positive throat cultures for haemolytic streptococci and GAS, the association of positive cultures with clinical symptoms of an upper respiratory tract infection and the variations in antibody responses between symptomatic and asymptomatic children with positive cultures. | Cohort | 248 children aged 8-10 from three schools  Nashville, USA | Socioeconomic status based on neighbourhoods where school were located | Throat swab culture for haemolytic streptococci and GAS |  | **Positive association**  Greater proportion of positive GAS swabs from 2 low socioeconomic schools compared to low-middle to middle class school (p<0.05) |  | Poor: poor description of socioeconomic measure, limited analysis. |
| Agarwal et al 1995 | To draw out the real picture of RHD prevalence in the general population of rural India. | Cross section | 3,760 people from 11 villages  Uttar Pradesh, India | Socioeconomic status based on Prasad classification of I to IV (I=highest). | RHD diagnosed through clinical examination screen, with investigation of ECG, CXR, cardiologist review, echocardiogram | 6.4/1000 | **Positive association**  RHD prevalence by SES: I: 0, II: 33.1/1,000, III 14.4/1,000, IV 4.5/1,000, V 4.8/1,000 (χ^2^=18.3, P<0.01) |  | Poor: poor description of methods, no power calculation. |
| Beaton et al 2012 | To use clinical examination and echocardiography to screen Ugandan school children to compare the yield of echocardiography and auscultation, evaluate the prevalence of RHD by sociodemographic criteria and assess the usefulness of laboratory data. | Cross section | 4,869 school students aged 5-16 years.  Uganda | Low, Middle, High socioeconomic status assigned according to school attended by participant based on location, tuition and estimated average household income. | Definite, probable, possible RHD diagnosed according to the 2006 WHO/NIH joint criteria. | 14.8/1000 | **Positive association**  RHD (any) prevalence in Low SES 2.79% vs. Middle SES 1.25% and High SES 1.44%)  RR 2.10 (1.18-3.74, p=0.01)  **my stat |  | Poor to fair: No power calculation, no multivariate, poor description of assignment of SES. |
| Bhave et al 1991 | To determine ASO antibodies in normal children compared with patients of ARF; to assess ASO antibodies in children suffering from clinically diagnosed streptococcal sore throat and correlate with bacteriological cultures of the throat, and to find out the point prevalence of RHD in children under 15 years | Cross section | Health records of 51, 992 school children reviewed.  Bombay, India | High SES: children who attended private schools  Low SES: children who attended free municipal schools | Diagnosis of RHD based on physicians’ diagnosis and treatment with aspirin/steroids. ARF diagnosed using radiological and electrocardiographical criteria.  Positive ASOT= titre>200IU/mL | RHD 1.5/1000 | **Positive association**  RHD prevalence in Low SES 0.17% vs. high SES 0.05% (p<0.05)  **No association**  ASOT and SES |  | Poor: poor description of design, exposure/outcomes measures. |
| Chaikitpinyo et al 2014 | To determine the epidemiological trend of ARF/RHD and its association with changes in SES in school children aged 5-15 years in Khon Kaen using the same screening method as most recent survey in area from 1986 | Cross section | 8,555 school children aged 5-15 year  Khon Kaen, Thailand | SES of school where child was attending | ARF diagnosed using Jones criteria  RHD diagnosed using examination by 2 cardiologists, and confirmed with ECG and echocardiography | ARF & RHD 0.23/1000 | **No association**  ARF or RDH and SES |  | Fair: small numbers, thorough methods. |
| Faruq et al 1995 | To detect the status of βHS infection in the throat of children and the role of environmental factors in the infection. | Cross section | 601 children aged 5-15 years  Dhaka, Bangladesh | Social status determined by possession of Video cassette recorder/player and private car. | Throat culture for βHS, GAS | 22% βHS +  4.2% GAS + | **No association**  βHS and SES  GAS and SES |  | Poor: non-participation not reported, no multivariate analysis. |
| Hammon et al 1950 | A preliminary study to explore the usefulness of a test to indicate past infection with poliomyelitis and the disease’s possible correlation to streptococcal infections. | Cross section | 653 school children aged 1-15  California, USA | Economic groups: low, high (not defined further) | ASOT | Various | **No association**  ASOT and SES group |  | Poor: inadequate description of methods. |
| Imamoglu et al 1988 | To assess the effect of socioeconomic state on the prevalence of RHD and whether there was any decline in the morbidity rate over the last decade with the improvement of medical facilities. | Cross section | 3,039 children  Ankara, Turkey | Socioeconomic group: Low group from shanty town, high group not defined | RHD diagnosed by screening by cardiologist and confirmed by electrocardiography, radiography and echocardiography | 6.58 /1000 | **Positive association**  RHD prevalence SES Low 1% vs. High 0.2% (p<0.01) |  | Poor: poor description of exposure measure, limited analysis. |
| Longo-Mbenza et al 1998 | A study of the prevalence of RHD in children of Kinshasa using echocardiography to confirm the diagnosis and to verify the possible relationship between host, slum environment and RHD. | Cross section | 4,848 students aged 5-16 years  Kinshasa, Democratic Republic of Congo | Urban vs. slum school  Socioeconomic status within slum: Low, High based on Prasad classification of family income | RHD diagnosed on echocardiography of suspect cases. | 14.03/1,000 | **Possible association**  RHD prevalence in slum students 22.2/1000 vs. urban students 4/1000  (no test of significance) | **Positive association**  Low SES OR 2.68 (1.43-5.01) | Fair: limited results presented. |
| McLaren et al 1975 | An epidemiological survey to define the exact magnitude of the problem of RHD in black children in the crèches and schools of Soweto. | Cross section | 12,050 black children aged 2-18 years  Soweto, South Africa | SES based on home assessment (not defined) | RHD diagnosed by clinical examination.  Throat swab culture for GAS | 6.9/1000 | **No association**  SES |  | Poor: outcome ascertainment method poor, no multivariate analysis. |
| Periwal et al 2006 | To use clinical and echocardiographic criteria to determine the prevalence of RHD in school children in a north-west Indian town | Cross section | 3,002 children aged 5-14 years | SES assigned according to school where child attended: High (Private school), Middle (High SES government school), Low ( Low SES government school) | Revised Jones criteria used for diagnosis of ARF and clinical and echocardiographic criteria for diagnosis of chronic RHD | Clinical RHD 16.7/1000  Echocardiographic RHD 0.67/1000 | **Positive association**  Clinical RHD prevalence Low SES 28.2/1000 vs. Middle SES 17.0/1000 and High SES 5.8/1000 (χ^2^ 175.2, p<0.0001) |  | Poor: large discrepancy in case ascertainment between methods not explained, no description of SES measure. |
| Poppi et al 1953 | To ascertain the prevalence of ARF and RHD in a region of Italy where the high incidence of both complaints is well known to practicing physicians, and to evaluate the weight of some factors generally admitted as important in the pathogenesis of the disease. | Cross section | 930 female manual labourers aged 14-70 years  Po valley, Italy | Type of work: Rice worker proxy for lowest economic standards and living condition compared to other labourers. | ARF based on clinical history of acute migrating polyarthritis, confining patient to bed with fever and subsiding after salicylates, or of Sydenham’s chorea.  RHD diagnosed with examination, orthodiagram of the heart and ECG. | 14.9% history of ARF or chorea  8.7% RHD | **Positive association**  ARF prevalence in Rice workers 24.3% vs. Other labourers 6.8%, RR 3.72 (2.4 - 5.76)  RHD Rice Workers 11.3% vs. Other labourers 2.8%, RR 3.76 (1.9—7.45)* |  | Poor: ill-defined exposure and poor presentation of results. |
| Quinn et al 1948 | To select and examine comparable rural and urban populations from a single small area of the USA and to determine the rates for RHD in each population, and to answer: what is the effect of living conditions within high and low rental areas in cities; what affect does crowding in the home have on the prevalence of RHD; and what is the familial incidence of ARF and RHD in this geographic area. | Cross section | 3,141 children rural, semi-urban and urban based, aged 11-15 years  Connecticut, USA | Type of rental area where home is located:  Low, medium, high | ARF history based on clinical history of rheumatic fever or chorea.  RHD diagnosed using criteria found on clinical examination | 2.1% history ARF  2.1% RHD | **No association**  Rental area |  | Poor to fair: Poor case ascertainment method, no multivariate analysis. Well described study. |
| Rizvi et al 2004 | To determine the prevalence of RHD in a rural population in a single subdistrict and study the risk factors for RHD. | Cross section | 10,412 participants interviewed & 9,483 screened across  11 rural villages  Pakistan | Socioeconomic score calculated from cost of items owned by family | ARF diagnosed using updated 1992 Jones criteria  RHD diagnosed using echocardiography for cases with clinical murmurs | RHD 5.7/1000 | **No association**  Socioeconomic score |  | Fair to good: generally good methods, unconventional measure for crowding index. |
| Saxena et al 2011 | To estimate the prevalence of clinical and subclinical RHD, to identify risk factors associated with RHD and to study the natural history of children with echocardiographically detected RHD. | Cross section | 6,270 school children aged 5-15 years  Rural area,  northern India | Type of school: Government (low) or private (high) | RHD diagnosed using modified WHO criteria of echocardiogram. | 20.4/ 1000 | **Positive association**  Government school 32.49/1000 vs. Private school 15.15/1000 (p<0.001) | **Positive association**  Government school OR 1.55 (1.02-2.34) | Fair: no power calculation. |
| Thakur et al 1996 | To undertake a school based study to investigate the current extent of RHD in Himachal Pradesh | Cross section | 15,080 school children aged 5-16 years  Shimla Hills, northern India | Type of school: Rural government, Urban government, Convent, Private  Social classes I-V using Modified Prasad Classification based on family income (I=highest) | ARF diagnosed using modified Jones criteria  Probable RHD diagnosed after screening, evaluation by cardiologist, Doppler echocardiography | RHD 2.92/1000  ARF 0.19/1000 | **Positive association**  ARF & RHD prevalence in Schools: Rural government 4.8/1000, Urban government 2.57/1000, Private 1.62/1000 Convent 1.6/1000, χ^2^=9.94 (p<0.05)  **No association**  Social class |  | Fair: well designed, large study, no power calculations or multivariate analysis |
| Yazov et al 1978 | To present the results of the first stage of a prospective epidemiological study of GAS and RHD. | Cross section | 1,012 school children aged 6-20 years  Addis-Ababa, Ethiopia | Social status assigned as either “Well to do” or “Poor” based on parents’ income | RHD diagnosed on clinical findings.  Throat swab culture | GAS incidence 4.24%    RHD prevalence 4.9/1000 | **No association**  GAS or RHD and Social status |  | Poor: poor description of methods and analysis. |
| Atatoa-Carr et al 2008 | To collect 7-year incidence and epidemiology of ARF in the Waikato DHB region, including assessing the recurrence rate of ARF without an organised programme and to explore further opportunities for ARF prevention and management in the Waikato region. | Ecologic | 77 cases ARF aged 4-32 years  Waikato, New Zealand | New Zealand Index of Deprivation (NZDep01) used as measure of deprivation of the address where each case resided at time of diagnosis. | ARF diagnosed using Jones criteria with New Zealand modifications | 3.3/100,000 | **Possible association**  78% in most deprived 3 deciles, 94% in most deprived 5 deciles  (no test of significance) |  | Poor to fair: no test of significance, no description of ecological units |
| Ferguson et al 1991 | To construct an epidemiological profile of ARF in a multiracial, multiethnic metropolis from 1984 through 1988. | Ecologic | 29 ARF cases | Socioeconomic status of minor statistical area | ARF diagnosed using revised Jones criteria | 3/100,000 for 5-14 year olds | **Possible association**  62% cases from 4 areas of lower SES  (no test of significance) |  | Poor: poor methods for ascertaining and reporting population and exposure measures |
| Gordis et al 1969 | To explore the relationship of socio-economic status to incidence rates of rheumatic fever to determine whether socio-economic differences can adequately account for ethnic differences in incidence. | Ecologic | Not reported.  Aged 5-19 years  Baltimore, USA | Census tracts divided to 5 socioeconomic categories (1=lowest) based on median rental stratified by race (white, non-white) | ARF diagnosed using hospital medical records between 1960 and 1964 | Various. From 3.4 per 100,000 among whites in highest socioeconomic fifth to 26.6 per 100,000 in blacks in lowest socioeconomic fifth. | **Possible association**  Average annual ARF incidence/100,000 in SES for white populations   1. 15.8 2. 14.2 3. 11.6 4. 8.0 5. 3.4   (no test of significance)  **No association**  Average annual ARF incidence and SES for non-white populations |  | Fair: limited factors explored, no multivariate analysis. |
| Holmes et al 1953 | To investigate the distribution of cases of childhood ARF in the different social classes in Melbourne and attempt to relate this to the incidence of streptococcal infection | Ecologic | 1,469 cases ARF in children up to 14 years over a 10 year period  Melbourne, Australia | Local government districts classified as high, medium and low rental areas | ARF diagnosed from hospital records | Average incidence 1.18/1000 | **Positive association**  ARF incidence in low rental areas 942/62,761, medium 325/40,309, and High 202/42,656.  Low vs. Medium/high RR 1.96 (1.77-2.18)* |  | Poor: limited factors included, not robust case ascertainment, no time trend analysed, no test of significance. |
| Holmes et al 1953 | To investigate the distribution of cases of childhood ARF in the different social classes in Melbourne and attempt to relate this to the incidence of streptococcal infection | Ecologic | 530 throat swabs from three schools aged 7-11 years | Children assigned social class based on location of school in low, medium, high rental areas | Throat swab for GAS | 12.5%-25.3% carrier rate | **Positive association**  GAS carrier rate in Low rental 25.3%, Medium rental 18.6%, High rental 12.5%, χ^2^=22.89 (p<0.001) |  | Poor: limited study. |
| Jaine et al 2011 | To test the hypothesis that household crowding was positively associated with ARF incidence and whether there was a dose-response relationship between the exposure and ARF risk. | Ecologic | 1,249 ARF cases between 1996 and 2005.  New Zealand | Cases assigned to a deprivation quintile of their census area unit derived from a composite measure of 9 variables from the New Zealand census (NZDep2006) (1= least deprived) | ARF diagnosed from hospital recorded diagnosis | Average annual rate ARF 3.4/ 100,000 | **Positive association**  Rate ratio of ARF cases /100,000 by deprivation quintile:   1. 1.0 (reference) 2. 1.7 (1.1-2.5) 3. 2.8 (2.0-4.1) 4. 5.0 (3.6-7.1) 5. 17.5 (12.7-24.3) |  | Good: well described study, consistent and valid exposure and outcome measures. |
| Milne et al 2012 | To estimate ARF incidence rates in detail with a focus on primary and intermediate school-age children (5 to 14 years of age) in order to inform targeting of a primary community intervention for school-age children such as sore throat clinics and/or an immunisation programme. | Ecologic | 1,552 ARF cases in children aged 5 to 14 years  New Zealand | Cases assigned to the deprivation decile of their census area unit derived from New Zealand Index of deprivation (NZDep2006) (1= least deprived) | ARF diagnosed from national dataset of hospital admissions with principle diagnosis of ARF | 17.2/100,000 | **Possible association**  Incidence rates for ARF increased with deprivation decile. 48% of cases in 10^th^ decile; 70% in 9^th^ & 10^th^ deciles (no test of significance) |  | Fair: validated exposure and outcome measures, large number limited analysis. |
| Pennock et al 2014 | The study was conducted as part of a national research project collecting retrospective data throughout New Zealand over a ten year period with the aim to gather concise, up-to-date epidemiological data. | Ecologic | 110 ARF cases  Waikato, New Zealand | Cases assigned to the deprivation decile of their census area unit derived from New Zealand Index of deprivation (NZDep2006) (1= least deprived) | ARF diagnosed from hospital admission database and EpiSurv database of national notifiable diseases, and confirmed using 1992 Jones criteria with New Zealand modifications | Total population 3.1/100,000/year  5-14 year olds 15.9/100,000/year | **Possible association**  73.1% cases lived in an area with deprivation decile of 7-10  (no test of significance) |  | Fair: validated exposure and outcome measures, small number limited analysis. |
| Quinn & Quinn 1951 | To study mortality rates due to RHD in New Haven between 1920-1948 by sociodemographic factors. | Ecologic | 1,144 RHD deaths  Conneticut, USA | Address at time of death located in one of three strata:  1: Best social indicators and incomes  2: Moderate incomes, better than “expected” social indicators  3: “Blighted” and slum areas. Below desirable and healthful standard housing, low income | RHD deaths ascertained from pre-defined list of deaths attributed to RHD on Death certificates |  | **Positive association**  RHD deaths/population by strata:   1. 143/34,633 2. 265/43,097 3. 680/84,551   Stratum 3 vs strata 1&2  RR 1.54 (1.36-1.74) * |  | Poor: consistent outcome measure, no statistical testing of trends, associations, use of address at time of death may precise reflection of exposure. |
| Quinn 1980 | To conduct a series of studies in samples of school children from Nashville, Tennessee between 1953-1954 and 1973-1974 to secure data on: 1) the incidence of haemolytic streptococci among young, school-aged children; 2) the significance of certain epidemiological factors; 3) the bacteriological characteristics of the isolated streptococci; 4) correlations between the acquisition of haemolytic streptococci with manifest clinical symptoms and antibody responses. | Ecologic | 53,827 throat cultures taken on 3,497 school children aged 5-13 years  Nashville, USA | Schools assigned socioeconomic status based on census tract: High, Middle, Low | Throat swabs for GAS, non-Group A streptococci | GAS 17.87%  Haemolytic streptococci 17.98% | **Positive association**  Difference in GAS positivity rates between children from low, middle and high socioeconomic schools (p<0.0001) (graphed data only) |  | Poor to fair: large numbers, but over time and trend analysis not included |
| Roberts et al 2015 | To describe the prevalence of definite and borderline RHD in Indigenous children in different regions of Australia and the Torres Strait and to inform decision making about the potential impact and usefulness of echocardiographic screening for RHD in different Australian regions. | Ecologic | 3,964 Indigenous children aged 5-14 years in four different regions  Australia | Australian Bureau of statistics indices:  Index of Community Socio-Educational advantage  Index of Relative Social Advantage and Disadvantage  Index of relative social disadvantage | RHD diagnosed using World Heart Foundation echocardiographic criteria for all participants. | Various. From 4.7/1000 in Far North Queensland to 15/ 1000 in Top End region. | **Possible association**  Region with significantly higher RHD prevalence had lowest socioeconomic indices. (No direct test of significance) |  | Poor to fair: low participation rate, no test of significance of SES factors and RHD prevalence. |
| Robin et al 2013 | To establish the accuracy and completeness of surveillance for ARF in Northland for the 10 year period 2002-2011 as a robust baseline for future comparison, given current prevention efforts. | Ecologic | 114 ARF cases  Northland, New Zealand | Case’s residence at time of diagnosis assigned to a deprivation decile based on the New Zealand census deprivation index (NZDep2006) | ARF cases identified through audit of hospital discharges and met criteria of 2008 Health Foundation guidelines. | Annualised incidence rate 7.7/100000 | **Possible association**  ARF cases and deprivation: 55% cases resided in most deprived decile; 89.5% cases resided in deciles 8-10  (No test of significance) |  | Fair: validated exposure and outcome measures, limited analysis. |
| Siriett et al 2012 | To establish current rates of ARF in the Tairawhiti DHB region and to identify areas where future ARF primary prevention programmes maybe targeted in the region. | Ecologic | 44 ARF cases aged 4 to 24 years | Case’s residence at time of diagnosis assigned to a deprivation decile based on the New Zealand census deprivation index (NZDep2006) | ARF cases identified from hospital admissions, treatment database, and notifiable disease database Diagnosed using 1992 Jones criteria adapted for NZ use. | Annual total incidence 7.6/100000 | **Possible association**  ARF and deprivation: 80% resided in area with deprivation score of 8-10  (No test of significance) |  | Poor to fair: small number, ecologic units not described, limited analysis. |
| Smith et al, 2011 | To identify and describe all children admitted with ARF to a tertiary paediatric hospital in Sydney over a 9 year period and to describe their demographics and clinical characteristics, management and short-term outcomes. | Ecologic | 26 cases of ARF or RHD in children <15 years  Sydney, Australia | Postcode of case ranked according to the Socio-economic index for Area quintiles | Diagnosed ARF using 2005 Australian guidelines for ARF | NA | **Possible association**  SES quintile of cases:   1. 50% 2. 19% 3. 23% 4. 8% 5. 0%   (No test of significance) |  | Fair: well described study, limited factors and analysis. |
| Wedum et al 1944 | To examine all hospital admissions of cases of rheumatic fever in Cincinnati between 1930-1940. | Ecologic | 517 ARF cases of all ages with or without RHD  Cincinnati, USA | Median monthly rental < $21.65 | ARF diagnosed from hospital admission records  High ARF prevalence census tract= census tracts with ≥10 cases per 100,000  Low ARF prevalence census tract= census tracts with <10 cases per 100,000 | NA | **Positive association**  Monthly rental <$21.65 in 25/33 high prevalence tracts vs. 1/70 low prevalence tracts (χ^2^=61.8) |  | Fair: criteria for diagnosis not reported, statistical methodology not robust |

*Test of significance calculated for systematic review from original study data

ARF: Acute rheumatic fever ASOT: Anti-streptolysin O titre βHS: Beta haemolytic streptococci CXR: Chest X-ray ECG: Electrocardiogram GAS Group A streptococci NA: Not applicable OR: odds ratio RHD: Rheumatic heart disease SES: Socioeconomic status RR: Risk ratio UK: United Kingdom USA: United States of America WHO: World Health Organization
